# Harnessing Immune Rejuvenation: Advances in Overcoming T Cell Senescence and Exhaustion in Cancer Immunotherapy

**DOI:** 10.1111/acel.70055

**Published:** 2025-04-03

**Authors:** Tesfahun Dessale Admasu, John S. Yu

**Affiliations:** ^1^ Department of Neurosurgery Cedars‐Sinai Medical Center Los Angeles California USA; ^2^ Kairos Pharma Los Angeles California USA

**Keywords:** CAR‐T, immune checkpoints, immune rejuvenation, immunosenescence, immunotherapy, senolytic therapy, solid tumors, T cell exhaustion, T cell senescence

## Abstract

Immunotherapy has transformed the landscape of cancer treatment, with T cell‐based strategies at the forefront of this revolution. However, the durability of these responses is frequently undermined by two intertwined phenomena: T cell exhaustion and senescence. While exhaustion is driven by chronic antigen exposure in the immunosuppressive tumor microenvironment, leading to a reversible state of diminished functionality, senescence reflects a more permanent, age‐ or stress‐induced arrest in cellular proliferation and effector capacity. Together, these processes represent formidable barriers to sustained anti‐tumor immunity. In this review, we dissect the molecular underpinnings of T cell exhaustion and senescence, revealing how these dysfunctions synergistically contribute to immune evasion and resistance across a range of solid tumors. We explore cutting‐edge therapeutic approaches aimed at rewiring the exhausted and senescent T cell phenotypes. These include advances in immune checkpoint blockade, the engineering of “armored” CAR‐T cells, senolytic therapies that selectively eliminate senescent cells, and novel interventions that reinvigorate the immune system's capacity for tumor eradication. By spotlighting emerging strategies that target both exhaustion and senescence, we provide a forward‐looking perspective on the potential to harness immune rejuvenation. This comprehensive review outlines the next frontier in cancer immunotherapy: unlocking durable responses by overcoming the immune system's intrinsic aging and exhaustion, ultimately paving the way for transformative therapeutic breakthroughs.

## Graphical abstract

Cancer can weaken the immune system by pushing T cells into exhaustion or permanent aging (senescence), making treatments less effective. This review explores groundbreaking therapies—like immune boosters, cell engineering, and senolytics—that aim to rejuvenate T cells, helping the body fight cancer more effectively and for longer.

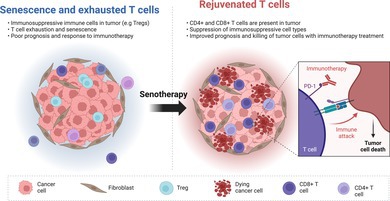

## 1 Introduction: Immunosenescence

**FIGURE 1:**
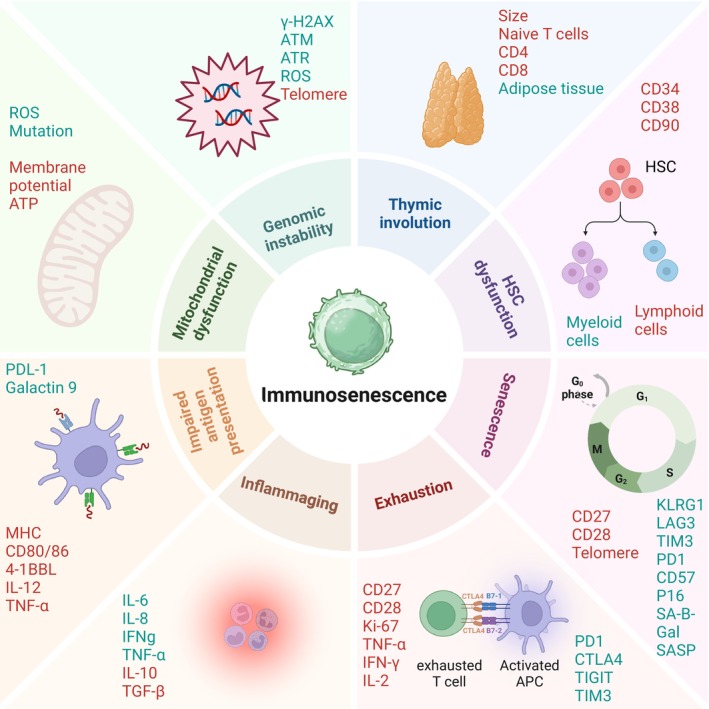
Hallmarks of Immunosenescence. The hallmarks of immunosenescence are summarized by thymic involution, HSC dysfunction, T cell senescence, exhaustion, chronic low‐grade inflammation, impaired antigen presentation, mitochondrial dysfunction, and genomic instability. HSC, hematopoietic stem cells. The image was created with BioRender (https://www.biorender.com/).

Aging profoundly impacts the immune system through a process known as immunosenescence, which is characterized by a gradual decline in both innate and adaptive immune function. This decline is closely linked to inflammaging—a state of chronic, low‐grade sterile inflammation that develops with age (Lian et al. 2020). Immunosenescence and inflammaging are driven by multiple factors such as persistent antigenic load, cellular damage, metabolic stress, and thymic involution (Liu et al. 2023). As early as young adulthood, the thymus—a key organ for generating naïve T cells—begins to shrink. Across age, thymic epithelial tissue is gradually replaced by adipose tissue, impairing the production of naïve T cells (Liang et al. 2022; Kousa et al. 2024). Additionally, there is an age‐related shift in hematopoietic stem cells (HSCs) toward myeloid cell differentiation at the expense of lymphoid lineages (Mejia‐Ramirez and Florian 2020). As a result, older individuals exhibit a diminished pool of naïve T cells, compromising the immune system's ability to mount effective responses (Ostrand‐Rosenberg and Sinha 2009; Ponnappan and Ponnappan 2011) (Figure 1). Importantly, cellular senescence represents a hallmark of aging and plays a pivotal role in driving these immune alterations. Senescent cells in the thymus and bone marrow secrete SASP factors, which exacerbate inflammaging and disrupt the microenvironment required for T cell development and renewal. These changes underscore the intricate connection between aging, cellular senescence, and immunosenescence.

## 2 T Cell Senescence

T cell senescence represents a specific aspect of immunosenescence, focusing on the functional decline of T cells. Following their release from the thymus, naïve T cells continuously patrol the body, scanning for antigens through their T cell receptor (TCR). Upon antigen recognition, T cells become activated through TCR engagement and co‐stimulation by CD28, which binds to B7‐1/B7‐2 on antigen‐presenting cells (APCs) (Han et al. 2023; Mellman et al. 2023). Once activated, T cells can follow one of three primary fates: apoptosis following antigen clearance, exhaustion after repeated low‐dose stimulation, or differentiation into long‐lived memory T cells (Turner et al. 2021). These memory cells include central memory T (Tcm) cells, which reside in lymphoid tissues and retain self‐renewal capacity, and effector memory T (Tem) cells, which reside in peripheral tissues and retain cytolytic functions despite reduced proliferative capacity (Martin and Badovinac 2018). Over time, repeated antigen exposure and aging lead to the accumulation of terminal effector T (TE) cells, known as TEMRA cells. These cells are characterized by the loss of CD27, CD28, and CCR7, along with the re‐expression of CD45RA (Wherry and Kurachi 2015). Although TEMRA cells are often considered as senescent T cells, their precise role in immunosenescence remains under investigation (Henson et al. 2015). In addition to the decline in naïve T cells, aging promotes the accumulation of senescent and exhausted T cells. Senescent T cells are characterized by an irreversible cell‐cycle arrest and the secretion of SASP factors, contributing to a pro‐inflammatory environment. Exhausted T cells, on the other hand, arise from chronic antigen stimulation and exhibit diminished effector functions such as reduced cytokine production and cytotoxicity. Both cell types contribute to immune dysfunction.

In conclusion, while immunosenescence encompasses the broader decline in immune function associated with aging, T cell senescence specifically highlights the deterioration within the T cell compartment. These age‐related changes contribute to the progressive deterioration of T cell‐mediated immunity and create a permissive environment for the growth of tumors. Therefore, interventions targeting T cell senescence and exhaustion hold promise for improving immune function and enhancing responses to immunotherapies in the context of cancer.

## 3 Senescent Cells in the TME

The TME is composed of tumor cells and several categories of non‐tumor cells such as fibroblasts, endothelial cells, and a variety of immune cells. From the immune cells, CD8+ T cells, CD4+ T cells, natural killer (NK) cells, M1 macrophages, and DCs have an anti‐tumor effect. On the other hand, MDCSs, Treg cells, and TAM have a protumor effect and suppress tumor immunity (Gajewski et al. 2013). Senescent cells in the TME have a contradictory role in tumor progression, which can either promote or suppress cancer. Cellular senescence acts as a tumor‐suppressive mechanism by restricting the proliferation of damaged cells, including those with activated oncogenes, which are known to drive tumorigenesis (Coppé et al. 2010; He and Sharpless 2017). Spontaneous and therapy‐induced senescence (TIS) inhibits tumor growth and stimulates anti‐tumor immune responses through several mechanisms, including immune cell recruitment (DCs, NK cells, and T cells) to the TME, activation of TILs, and antigen presentation (Ruscetti et al. 2018, 2020). Similarly, SASP factors such as IL‐6, IL‐8, CCL5, and CXCL1 are known to promote immune surveillance (Faget et al. 2019) by recruiting M1‐like macrophages, NK cells, and CD8 T cells to the TME (Ruscetti et al. 2020). Senescent cells can also present antigens to the immune cells. They are characterized by an elevated expression of MHC‐I together with the molecular machinery required for the processing and presentation of antigens, rendering senescent cells highly sensitive to recognition and killing by T cells (Marin et al. 2023; Chen et al. 2023a; Gilioli et al. 2022). Indeed, senescent cancer cells produced a stronger immunization than cancer cells undergoing immunogenic cell death. In addition to directly boosting the immunogenicity of target cells, the induction of senescence in neoplastic cells also enhances T cell‐dependent surveillance by promoting the recruitment and maturation of DCs (Marin et al. 2023).

**FIGURE 2:**
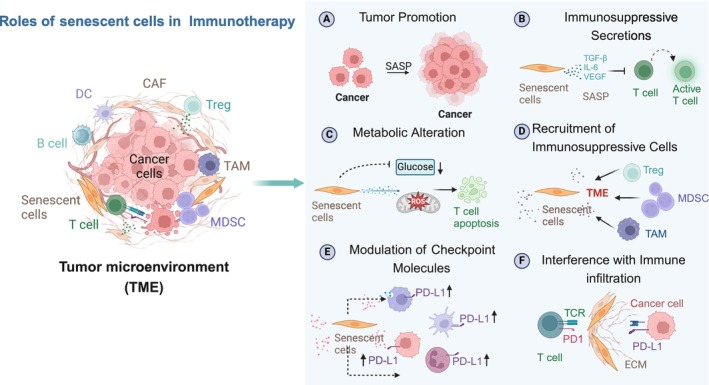
Role of senescent cells in cancer Immunotherapy. This figure illustrates the multifaceted role of senescent cells and the SASP factors secreted by senescent cells in the TME and their impact on cancer immunotherapy. SASP, senescence‐associated secretory phenotypes. The image was created with BioRender (https://www.biorender.com/).

However, in other settings, senescent cells have been shown to contribute to the generation of a protumor and immune‐suppressed microenvironment, thereby promoting tumorigenesis (Figure 2). Senescent cells can be tumor‐promoting through the secretion of immune suppressive factors, the attraction of immune suppressive cell types, as well as the production of angiogenic and other growth factors (Coppé et al. 2010; Ruscetti et al. 2020; Demaria et al. 2017; Gonzalez‐Meljem et al. 2018). For example, senescent endothelial cells create an immunosuppressive TME by favoring tumor infiltration of Tregs and MDSCs and inhibiting infiltration of CD8 T cells, DCs, and NK cells. The SASP factors may also be important mediators of the pro‐tumorigenic effects of senescent cells by creating a chronic inflammatory microenvironment that supports cancer growth (Coppé et al. 2008; Lasry and Ben‐Neriah 2015). For example, IL‐6 secreted by senescent cells in the TME increases the number of tumor‐infiltrating MDSCs and suppresses the function of T cells (Faget et al. 2019; Ruhland et al. 2016).

Further, senescent cells in the TME are known to express inhibitory ligands and are resistant to immune‐mediated clearance. For example, therapy‐induced senescent cells have upregulation of PD‐L1 and PD‐L2 (Reimann et al. 2021; Chaib et al. 2022; Shahbandi et al. 2022) and anti‐PD‐L2 treatment is strongly synergistic in combination with standard senescence‐inducing chemotherapy in mice, acting as a senolytic immunotherapy (Wang et al. 2022a). Recent works also showed that senescent cells with high levels of PD‐L1 inhibit the cytotoxic function of CD8 T cells (Wang et al. 2022a; Onorati et al. 2022). These contradictory effects of senescent cells illustrate the complexity of their functions in the TME and the need to dissect the roles of specific senescent cell subsets and their network of interactions.

The standard cancer treatments—radiation, chemotherapy, and surgery—often result in TIS, contributing to the accumulation of senescent cells that can influence immunotherapy outcomes. Evidence from studies in several types of tumors suggests that the accumulation of senescent cells interferes with immunotherapy, and their removal can reverse this phenotype. For example, recent studies in mice have demonstrated that TIS causes resistance to immunotherapy by reducing CD8+ T cell infiltration and activation within tumors, leading to poor therapeutic outcomes (Demaria et al. 2017; Ruhland et al. 2016). Senescent cells can also synergize with the immunosuppressive microenvironment of tumors. Specifically, they may interact with myeloid cells, which limits the accumulation of CD8+ T cells in the TME and diminishes the efficacy of immunotherapies (Weber et al. 2018; Milanovic et al. 2018). Furthermore, even when a large number of TILs are present in the TME, tumors remain aggressive, suggesting that senescent cells impair effective anti‐tumor responses and immunotherapy (Rao and Jackson 2016; Kazemi et al. 2022). Additionally, senescent cells in the TME may promote T cell senescence through SASP factors or direct contact (Rao and Jackson 2016; Hernandez‐Segura et al. 2018). Therefore, the presence of senescent T cells poses significant challenges for tumor immunotherapy, which will be discussed next.

Notably, these mechanisms do not manifest uniformly across all cancers and can vary depending on the organ system or disease context. For instance, in the CNS, the immune‐privileged environment may dampen senescence‐associated immune activation, altering the outcomes of senescent cell accumulation. Similarly, primary tumors and metastatic lesions often exhibit distinct TME compositions, which may influence the role of senescent cells in shaping immune responses and immunotherapy outcomes.

## 4 Mechanism of T Cell Senescence in the TME

Immunosenescence associated with aging reflects a gradual decline in T cell function due to thymic involution and metabolic stress. In contrast, pathological senescence in the TME is driven by tumor‐specific factors such as DNA damage, oxidative stress, telomere attrition, chronic antigen stimulation, and inflammatory signals. Particularly in solid tumors, T cell senescence may be exacerbated by the immunosuppressive nature of the TME, which is characterized by inhibitory immune checkpoints, metabolic constraints, and the presence of immunosuppressive cell populations such as Tregs, TAM, and MDSCs (Woroniecka et al. 2018).

The primary suspects for driving T cell senescence in the TME are nutritional competition, dysregulation of nutrient signaling, and the metabolic reprogramming of T cells (Zhao et al. 2020). While naïve T cells rely on oxidative phosphorylation and fatty acid oxidation to provide energy, upon recognizing an antigen, naïve T cells are activated and differentiate into effector T cells (Sasidharan Nair et al. 2021; Zhang et al. 2024). During this process, mTOR signaling enables a metabolic switch from oxidative phosphorylation to aerobic glycolysis (Fox et al. 2005; Vander Heiden et al. 2009). This shift supports the increased glucose demand in the TME, where competition for glucose among T cells and tumor cells can impact T cell activation, proliferation, and effector functions. Interestingly, SCs are also known to be highly dependent on glycolysis, which all leads to metabolic competition and stress. This metabolic competition results in insufficient energy for T cells and impairs T cell function, proliferation, survival, and promotes senescent phenotypes (Liu et al. 2018; Xia et al. 2021). In association with this, inadequate blood supply to the tumor leads to hypoxic conditions and an acidic environment. Hypoxia and acidic pH conditions are hallmarks of the TME, playing crucial roles in tumor development. Hypoxia and acidic pH are known to cause senescence in different types of cells, including immune cells (Schito and Semenza 2016; Jing et al. 2019).

The other main cause of T cell senescence in the TME is Telomere Shortening. According to the tumor immune cell cycle, T cells, once they recognize their antigen, undergo extensive proliferation, which may lead to telomere attrition (Mellman et al. 2023; Zhao et al. 2020). However, it is important to note that T cells differ from other somatic cells in the expression of the enzyme telomerase. When a T cell recognizes its antigen, TCR activation leads to a transient upregulation of telomerase (Hodes et al. 2002) which enables clonal expansion of T cells and long‐lived immunity (Akbar and Vukmanovic‐Stejic 2007; Rudolph et al. 1999). However, loss of CD28, which is required for telomerase induction, results in lower telomerase activity and telomere shortening. The most striking finding in this regard is that T cells can elongate their telomeres, even when they do not express telomerase themselves, by acquiring telomeres from vesicles released by APC when they form an immunological synapse (Lanna et al. 2022). Nevertheless, TERT‐transduced T cells are still susceptible to telomere‐independent senescence, which possibly results from unrepaired cumulative DNA damage that ultimately leads to growth arrest (Scharping et al. 2016). This brings us to the third cause of T cell senescence: DNA damage. As T cells migrate toward sites of TME, they are likely to be subjected to high amounts of ROS generated by innate immune cells such as neutrophils and macrophages. This may cause DNA damage and subsequently senescence. Further, chronic antigenic stimulation induces DNA damage, activating tumor suppressor pathways p53 and p16 to halt the cell cycle and prevent mutation propagation. However, continuous proliferation increases mutation risk, driving genomic instability and senescence (Liu et al. 2023; Campisi 2013).

The TME is characterized by a high presence of immunosuppressive cells, such as TAMs and Tregs. Additionally, in the early stages of tumor formation, adaptive immune cells migrate to the tumor site, leading to dysregulation of cytokines and chemokines, which in turn promotes an immunosuppressive phenotype and contributes to the induction of senescence (Hambardzumyan et al. 2016). Aged macrophages cause paracrine senescence through the secretion of extracellular vesicles (Hou et al. 2024). Similarly, TAMs release reactive oxygen species (ROS), nitric oxide (NO), interleukin (IL)‐10, and transforming growth factor (TGF)‐β, all known to drive senescence (Dehne et al. 2017). Furthermore, TAMs facilitate the conversion of T cells into Tregs within the TME and enhance the recruitment and immunosuppressive activity of Tregs, which not only impair cytotoxic T cell function but also induce senescence through factors like IL‐10 and TGF‐β (Gabrilovich and Nagaraj 2009). In a first‐in‐human study of epidermal growth factor receptor variant III (EGFRvIII)‐specific CAR‐T cells for glioblastoma (GBM), analysis of tumor specimens from patients who underwent surgery post‐treatment revealed an influx of Treg cells, potentially limiting the anti‐tumor effects of CAR‐T therapy (Rourke et al. 2017). Therefore, elevated levels of TAMs and Tregs may synergistically inhibit T cell‐mediated anti‐tumor immunity by inducing senescence in the TME.

The other important cause of T cell senescence in the TME is cancer and other senescent cells. Almost all types of cancer therapies, including chemotherapy, radiation, and surgery, are known to cause senescence in the TME known as TIS (detailed review elsewhere; Schmitt et al. 2022; Sullivan et al. 2024). TIS cells accumulated after chemotherapy may induce paracrine senescence in the immune cells in the TME through direct contact or by releasing SASP factors. For example, in mice, CAF senescence limits the presence of activated CD8 + T cells in the TME, and the removal of senescent CAFs increases activated CD8+ T cells and potentiates immune checkpoint therapy (Assouline et al. 2024). Further depletion of CAF reduces the number of Treg cells and reduces tumor growth (Mhaidly and Mechta‐Grigoriou 2021). Another study showed that the transfer of senescent T cells to young mice causes paracrine senescence in the recipient mice T cells caused by direct contact with the injected senescent cells or through SASP factors secreted by the injected senescent cells (Callender et al. 2018; Gasek et al. 2021).

## 5 Dissecting T Cell Senescence and Exhaustion

**FIGURE 3:**
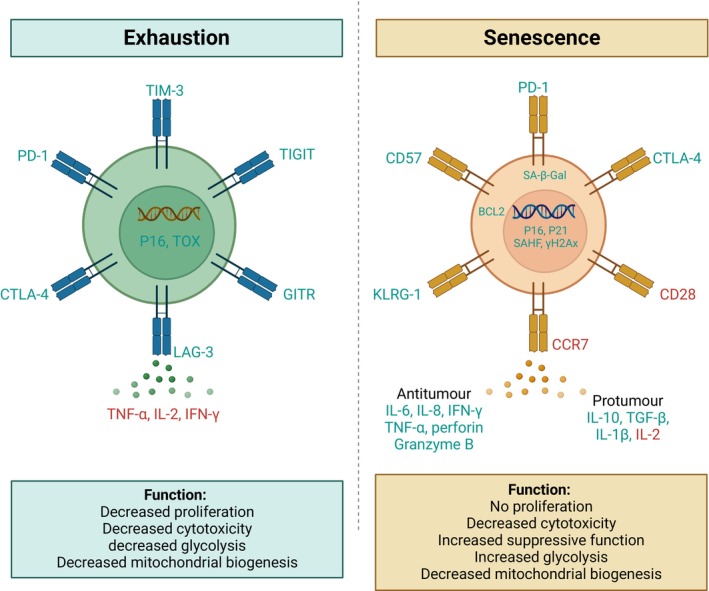
Comparison of T cell exhaustion and senescence. Unique and common markers of senescent and exhausted T cells and their functions are summarized. The image was created with BioRender (https://www.biorender.com/).

Both exhausted and senescent T cells share functional deficits and exhibit overlapping molecular features, making these two states difficult to distinguish (Figure 3). However, while exhausted and senescent T cells are often confused, recent findings indicate that they are distinct states of dysfunction (Slaets et al. 2024) and the fate of T cells exhaustion or senescence may be determined by a combination of intrinsic factors and signals from the TME. For example, aged CD8+ T cells intrinsically tend to be more prone to exhaustion, but the aged TME can induce these cells to adopt a senescence‐like state (Chen et al. 2024). Interestingly, the aged TME has been shown to push even young, transferred CD8+ T cells toward senescence (Chen et al. 2024), underscoring the critical role of signals from the TME in determining T cell fate.

Due to chronic antigen exposure in persistent tumors, T cells undergo a state of exhaustion. T cell exhaustion is characterized by impaired effector functions, reduced cytokine production, diminished cytotoxicity, expression of inhibitory receptors, including PD‐1, LAG‐3, TIM‐3, CTLA‐4, and TIGIT, and loss of proliferative capacity (Saleki and Rezaei 2022; Chow et al. 2022). Unlike senescent T cells, exhausted T cells do not show high levels of senescence‐associated β‐galactosidase (SA‐β‐Gal) activity (Janelle et al. 2021) and have decreased glycolysis (Han et al. 2023; Cao et al. 2023) (Figures 1 and 3). Further, exhausted T cells are not in a state of irreversible arrest and can potentially regain function with ICIs.

Senescent T cells are characterized by the loss of proliferative capacity, high levels of SA‐β‐Gal activity, and upregulated cell‐cycle regulators like p16 and p21 (Hernandez‐Segura et al. 2018). These cells may also upregulate NK cell‐like receptors, including killer cell lectin‐like receptor subfamily G Member 1 (KLRG1) and CD57, and lose cytotoxic capabilities, such as decreased production of granzyme B and perforin (Liu et al. 2023; Zhang et al. 2021). They also exhibit loss of C‐C chemokine receptor type 7 (CCR7), re‐expression of CD45RA, and the absence of costimulatory molecules such as CD28 or CD27 (Huang et al. 2024; Kunert et al. 2019). These cells exhibit impaired effector functions, including cytokine production and cytotoxicity, while paradoxically contributing to the pro‐inflammatory milieu (Covre et al. 2020; Liu et al. 2020a). Additionally, they often accumulate dysfunctional mitochondria and resist apoptosis through the upregulation of BCL‐2 (Hu et al. 2022).

The prevalence of exhausted and senescent T cells varies across tumor types and patient demographics. Exhausted T cells are more commonly associated with solid tumors due to chronic antigen stimulation, whereas senescent T cells are frequently observed in hematological malignancies (Jiang et al. 2015; Kasakovski et al. 2018). Hot tumors, such as melanoma, are characterized by higher infiltration of exhausted T cells. In contrast, cold tumors like GBM exhibit fewer T cells but a higher proportion of senescent immune cells (Woroniecka et al. 2018; Jiang et al. 2015; Miller et al. 2019; Liu et al. 2020b). Evidence suggests that advanced age is associated with higher levels of both exhausted and senescent T cells in tumors, likely due to immunosenescence and cumulative antigen exposure. However, senescence markers appear more pronounced than exhaustion in older patients (Kasakovski et al. 2018). Older patients exhibit phenotypic differences compared to younger patients, including an increased proportion of late‐differentiated T cells expressing markers of exhaustion, such as PD‐1, and senescence, such as the loss of CD28. These age‐related changes in T cell subsets and functionality impair the immune response to tumors and may influence the efficacy of immunotherapies, highlighting the importance of considering patient age in therapeutic strategies (Han et al. 2023; Fernandes et al. 2022; Yu et al. 2025). Although these trends are informative, they are based on limited evidence from small‐scale studies and tumor‐specific investigations. Variability in methodologies and markers used further complicates comparisons, highlighting the need for broader studies to clarify how the TME, treatment history, and patient age influence these T cell subsets. Further, despite these insights, the precise mechanisms driving T cell exhaustion versus senescence remain poorly understood.

A critical question that remains is whether therapies designed to target T cell exhaustion, such as ICIs, are effective against T cell senescence. Emerging evidence suggests that while ICIs can reverse exhaustion, they may not be effective in counteracting senescence. For instance, a subset of senescent‐like CD8+ T cells that are associated with aged TME has been shown to resist ICIs (Chen et al. 2024), indicating that these senescent‐like cells represent a unique challenge in the context of immunotherapy. While ICIs can restore the function of exhausted T cells, the persistence of senescent T cells may limit the overall efficacy of these treatments. To improve immunotherapy outcomes, particularly in older patients, it is essential to unravel the specific molecular mechanisms that drive T cell exhaustion and senescence in the aged TME. Therapeutic strategies must be developed to target both dysfunctional T cell states, thereby enhancing the overall immune response and improving the long‐term efficacy of cancer immunotherapy.

## 6 Cancer Immunotherapy

**FIGURE 4:**
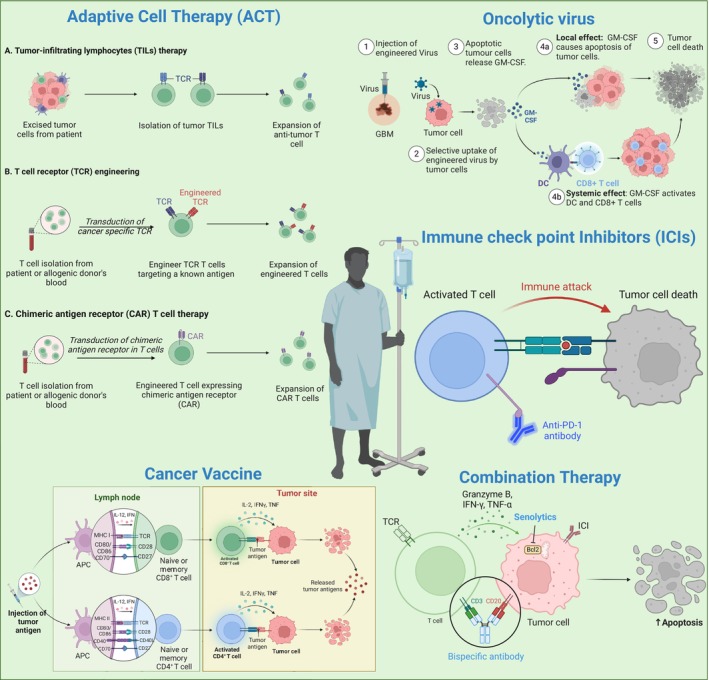
Current tumor immunotherapies. (i) adoptive T cell therapy, involving the infusion of (A) activated TILs or genetically modified (B) CAR‐T or (C) TCR‐T cells; (ii) cancer vaccine, which aims to activate the patient's adaptive immune system via the use of tumor‐specific or tumor‐associated antigens, delivered in the form of nucleic acids, peptides, or packaged into DCs; and (iii) oncolytic virus, which explores the use of viruses to selectively target and destroy cancer cells. (iv) Immune checkpoint therapy, utilizing monoclonal antibodies to remove the brakes on the immune system's response; (v) combination therapy, combining various immunotherapies for the treatment of tumors. CAR‐T, chimeric antigen receptor T; DCs, dendritic cells; TCR‐T, T cell receptor‐engineered T; TILs, tumor‐infiltrating lymphocytes. The image was created with BioRender (https://www.biorender.com/).

Cancer immunotherapy represents a paradigm shift approach that leverages the immune system's ability to recognize and eradicate tumors (Chen and Mellman 2017). Unlike conventional modalities such as chemotherapy and radiotherapy, which indiscriminately target both tumor and healthy cells, immunotherapies modulate immune pathways to enhance the immune system's ability to discriminate between self and tumor antigens (Mellman et al. 2011). A prominent class of these therapies involves T cell‐based strategies, which include immune checkpoint inhibitors (ICIs), CAR‐T cell therapy, TCR therapy, tumor‐infiltrating lymphocyte (TIL) therapy, oncolytic virus therapy, and Cytokine therapy (Figure 4). Together, these diverse T cell therapies have significantly improved outcomes in various cancer types, including melanoma, non‐small cell lung cancer (NSCLC), and renal cancer (Chen and Mellman 2013; Pardoll 2012; Sharma and Allison 2015a; Topalian et al. 2015).

However, the application of immunotherapy to solid tumors has been limited by the immunosuppressive characteristics of the tumor microenvironment (TME). The TME in solid tumors is characterized by multifaceted immune evasion mechanisms, including the accumulation of myeloid‐derived suppressor cells (MDSCs), regulatory T cells (Tregs), and tumor‐associated macrophages (TAMs) (Wherry 2011; Quail and Joyce 2013). Further, chronic exposure to tumor antigens within this hostile environment leads to T cell exhaustion, a state of progressive functional decline marked by the upregulation of inhibitory receptors such as TIGIT (T‐cell Immunoreceptor with Ig and ITIM domains), LAG‐3 (Lymphocyte‐Activation Gene 3), and TIM‐3 (T‐cell Immunoglobulin and Mucin‐domain containing‐3) (Wherry 2011). T cell exhaustion impairs the efficacy of immunotherapies by reducing T cell proliferation, cytokine production, and cytotoxicity of T cells (Pauken and Wherry 2015). In addition to exhaustion, senescence is another key mechanism contributing to immune dysfunction in the TME (Fu et al. 2023). Cellular senescence, originally described as a tumor‐suppressive mechanism, is increasingly recognized for its complex and multifaceted roles in tumor biology (Takasugi et al. 2022). Senescent cells are not merely passive bystanders but actively contribute to tumor progression and resistance to therapy (Woroniecka et al. 2018). Unlike exhausted T cells, senescent T cells undergo irreversible cell‐cycle arrest wherein they secrete pro‐inflammatory secretory phenotypes called senescence‐associated secretory phenotypes (SASP) (Fane and Weeraratna 2020). Senescent cells and the SASP factors further contribute to the immunosuppressive nature of the TME, exacerbating the challenge of achieving sustained anti‐tumor immunity (Takasugi et al. 2022). The other phenomenon is T cell quiescence, which is a state of reversible dormant state in naïve and memory T cells (Hamilton and Jameson 2012).

Addressing T cell exhaustion and senescence has become a focal point of recent immunotherapy developments. For example, dual checkpoint blockade, such as the combined inhibition of PD‐1 and LAG‐3, is being investigated to overcome multiple layers of immune suppression (Hodi et al. 2010). Advances in CAR‐T cell design, including dual‐targeting constructs and engineered resistance to exhaustion, are pushing the boundaries of adaptive cell therapy (ACT) in solid tumors (Bagley et al. 2024). Senolytic agents that selectively eliminate senescent cells offer the potential to dismantle the immunosuppressive signals within the TME and restore effective immune surveillance (Xu et al. 2018). In summary, while immunotherapy has revolutionized cancer treatment, understanding and targeting the complex mechanisms of immune evasion—particularly T cell exhaustion and senescence—within the TME is crucial for its success (Zingoni et al. 2024). This review will focus on the emerging prospects of tumor immunotherapy, with a particular emphasis on the roles of T cell exhaustion and senescence in modulating therapeutic outcomes.

## 7 Current Landscape of Tumor Immunotherapy

The key immunotherapeutic strategies under investigation include immune ICIs, cancer vaccines, ACT, oncolytic viruses, and combination therapies (Thomas et al. 2012) (Figure 4). Tumor immunotherapy continues to evolve, with an emphasis on overcoming immune evasion mechanisms, such as T cell exhaustion and cellular senescence, which impair the immune system's ability to eliminate tumors (Rong et al. 2022; Sharma et al. 2023).

### 7.1 ICIs

One of the landmark breakthroughs in cancer immunotherapy came with the development of ICIs, which target regulatory pathways in T cells to restore their anti‐tumor activity (Pardoll 2012). Tumors often exploit immune checkpoints like PD‐1 and CTLA‐4 to suppress immune responses and avoid detection (Wherry and Kurachi 2015). ICIs, such as nivolumab (anti‐PD‐1) and ipilimumab (anti‐CTLA‐4), have revolutionized cancer treatment by blocking these pathways, leading to the rejuvenation of exhausted T cells and enhanced anti‐tumor immunity (Topalian et al. 2015; Hodi et al. 2010; Sharma and Allison 2015b). However, the efficacy of ICIs is limited especially in some solid tumors due to factors like low neoantigen load and an immunosuppressive TME characterized by Tregs, MDSCs, and TAMs (Hodi et al. 2010; Zhao et al. 2019; Zamora et al. 2018).

### 7.2 Oncolytic Viruses

Oncolytic viruses are another innovative approach to tumor immunotherapy. These are genetically engineered or naturally occurring viruses that selectively infect and lyse tumor cells while simultaneously stimulating an anti‐tumor immune response (Kaufman et al. 2015). Oncolytic viruses can enhance immune recognition of tumors by releasing tumor‐specific antigens (TSAs) or tumor‐associated antigens (TAAs) and inducing the expression of immune‐stimulatory molecules (Asija et al. 2023; Russell et al. 2012). For example, T‐VEC expresses the immune‐stimulatory cytokine GM‐CSF, which promotes the activation of dendritic cells (DCs) and enhances the immune response (Andtbacka et al. 2015). These viruses can modulate the TME, reduce immune checkpoints, and promote immune cell infiltration (Ribas et al. 2017; Desjardins et al. 2018). However, challenges such as pre‐existing immunity to viral vectors and ensuring efficient delivery to tumor sites remain.

### 7.3 Cancer Vaccines

Cancer vaccines aim to train the immune cells to recognize and attack tumors by targeting specific antigens. These vaccines can be peptide‐based, DNA‐based, or DCs to present antigens to T cells. DC vaccines, in particular, hold promise due to their ability to induce strong anti‐tumor immune responses (Yamanaka et al. 2003). For example, the double‐blind randomized phase II trial of ICT‐107, a peptide‐pulsed DC vaccine targeting six GBM‐associated antigens, demonstrated a significant improvement in progression‐free survival (PFS) by 2.2 months compared to the control group receiving unpulsed DC. In this trial, HLA‐A2+ patients exhibited a stronger immune response and greater clinical benefit, particularly in the MGMT‐methylated subgroup (Wen et al. 2019). These findings highlight the potential of antigen‐specific DC vaccination in GBM treatment, warranting further investigation. The peptide‐based vaccine targeting EGFRvIII and a combination of a vaccine targeting survivin with temozolomide (TMZ) have shown extended survival benefits in early‐phase trials (Fenstermaker et al. 2016). Personalized mRNA vaccines offer a novel approach by tailoring the vaccine to the unique mutational profile of a patient tumor. These vaccines enable rapid and precise targeting of neoantigens, potentially overcoming challenges of antigen heterogeneity (Wilkinson 2023; Rojas et al. 2023). Despite some successes in early‐stage trials, challenges such as antigen heterogeneity, low immunogenicity, and antigen loss variants remain as barriers (Saxena et al. 2021; Grimmett et al. 2022; Katsikis et al. 2024; Lin et al. 2022).

### 7.4 Adaptive T‐Cell Therapy (ACT)

ACT involves the ex vivo expansion and reinfusion of tumor‐specific T cells into patients (Dudley and Rosenberg 2003; Rohaan et al. 2019; Posey et al. 2024). This approach includes TILs, T‐cell receptor (TCR)‐engineered T cells, and chimeric antigen receptor (CAR) T cells (Mohanty et al. 2019; Klebanoff et al. 2023). CAR‐T therapy, which engineers T cells to express receptors that recognize specific tumor antigens, has shown remarkable success in hematological malignancies. CAR‐T cells targeting TSAs such as EGFRvIII, IL‐13Rα2, and HER2 have also shown promising results in preclinical and early‐phase clinical trials in solid tumors (Morgan et al. 2012; Durgin et al. 2021; Brown et al. 2024). However, their application in solid tumors presents challenges due to antigen heterogeneity, poor T cell trafficking, and an immunosuppressive TME (Pant and Lim 2023). To address these challenges, strategies such as dual‐targeting CARs, armored CARs that secrete cytokines, and locally delivered CAR‐T cells are being developed (Bagley et al. 2024). Preclinical studies suggest that these next‐generation CAR‐T cells may overcome some limitations observed in previous trials (Young et al. 2022; Akhavan et al. 2019). TCR‐engineered T cells targeting NY‐ESO‐1 and other TAA are under evaluation in clinical studies, with the potential to enhance T cell specificity and function against tumor cells (Thomas et al. 2018; Everson et al. 2016). Lastly, trials on TILs indicate that the expansion of TILs isolated from solid tumors is less robust due to the inherently low immune cell infiltration. Further, enhancing the functionality and persistence of TILs within the TME remains a significant challenge (Liu et al. 2017; Verma et al. 2022; Watowich et al. 2023).

### 7.5 Combination Therapies

Combination therapy aims to target multiple aspects of tumor biology and immune evasion to mitigate resistance mechanisms, improve T cell persistence, and modulate the TME to overcome immunosuppressive barriers. Combining ICIs with conventional treatments (e.g., radiation, chemotherapy) or other immunotherapies (e.g., oncolytic viruses, CAR‐T cells) has shown promise in enhancing therapeutic efficacy (Rong et al. 2022; Acosta et al. 2013; Migliorini and Dutoit 2016). Radiation can increase tumor antigen release, while chemotherapy can reduce tumor burden, creating a more favorable environment for immune responses (Bausart et al. 2022; Maity et al. 2018). This effect is exemplified by the abscopal effect, where localized radiation not only induces tumor cell death at the treated site but also triggers systemic immune responses, leading to regression of distant, untreated tumors (Nabrinsky et al. 2022). For example, the combination of TMZ with the IMA950/Poly‐ICLC vaccine is currently being explored to leverage chemotherapy‐induced immunogenic cell death along with vaccine‐induced immune activation (Migliorini and Dutoit 2016; Todo et al. 2022; Migliorini et al. 2019). In addition, ICIs can reinvigorate T cells, while cancer vaccines can enhance local tumor lysis and stimulate a broader immune response (Antonios et al. 2016; Aghajani et al. 2024). For example, DCVax‐L, a personalized DC vaccine combined with standard therapies, and ATL‐DC, which is being tested in combination with ICIs, have shown promising results in recurrent GBM (Gatto et al. 2023; Everson et al. 2024). In a Phase III trial of DCVax‐L, the median overall survival (OS) was 23.1 months for newly diagnosed GBM patients and 13.2 months for recurrent GBM patients, as compared to historical controls. The lack of contemporaneous controls impacts the validity of this trial. Nevertheless, a favorable safety profile was also observed. ATL‐DC also boosted T cell activation and infiltration into the TME, countering GBM's immunosuppressive milieu. Its combination with ICIs has led to a favorable OS and increased tumor shrinkage compared to ICIs alone, highlighting its potential to synergistically enhance anti‐tumor immunity (Everson et al. 2024). Further, a multi‐epitope DC vaccine demonstrated a progression‐free survival benefit in a randomized placebo‐controlled trial in newly diagnosed GBM patients (Phuphanich et al. 2013).

In summary, recent clinical trials highlight both the promise and challenges of tumor immunotherapy (Weller and Le Rhun 2019). The field is moving toward combination strategies (Bausart et al. 2022), innovative vaccine platforms (Liau et al. 2023), and a deeper understanding of the TME (Sharma et al. 2023) to improve patient outcomes. Ongoing research and future trials will continue to refine these approaches, develop reliable biomarkers, and explore novel combinations to fully realize the potential of immunotherapy.

## 8 Addressing T Cell Exhaustion and Senescence to Enhance Immunotherapy

Overcoming T cell exhaustion and senescence is essential for improving outcomes in immunotherapy. ICIs have shown limited efficacy in solid tumors, partly due to the high prevalence of senescent T cells within the TME (Maggiorani et al. 2024). Similarly, ACT can be compromised by T cell senescence, leading to decreased therapeutic effectiveness (Amor et al. 2020). Oncolytic viruses and cancer vaccines, designed to boost T cell‐mediated anti‐tumor immunity, may also face challenges in the presence of these dysfunctional T cells (Chen et al. 2023a). Eliminating senescent cells has shown promise in restoring immune homeostasis and improving survival in preclinical models (Wang et al. 2022b; Prasanna et al. 2021). Therefore, understanding the role of T cell senescence in immunotherapy is crucial for developing more effective treatment strategies. In this section, we explore therapeutic approaches aimed at targeting these dysfunctional T cells.

### 8.1 Immunomodulatory Interventions

Immunomodulatory interventions, including ICIs, cytokine therapies, and vaccination strategies, aim to reverse T cell exhaustion and reinvigorate anti‐tumor immunity. Although ICIs revolutionized cancer treatment, single‐agent ICIs often offer limited benefits in highly immunosuppressive tumors like GBM (Chow et al. 2022; Rafiq et al. 2020). Consequently, combining ICIs with agents targeting inhibitory receptors expressed by senescent T cells, such as TIM‐3, LAG‐3, and TIGIT, has emerged as a promising approach to overcome resistance and enhance T cell rejuvenation (Lu and Tan 2024; Anderson et al. 2016). Recent studies have highlighted the potential of targeting PD‐L1/PD‐L2‐expressing senescent cells to enhance T cell‐mediated elimination of senescent cells in aging and cancer models. For example, anti‐PD‐1 therapy effectively increases the immune clearance of PD‐L1+ senescent cells in aging models (Onorati et al. 2022), suggesting a strategy to exploit ICIs against senescent cell populations within tumors. Similarly, targeting senescent cell‐specific markers alongside ICIs could restore anti‐tumor immunity in preclinical models (Wang et al. 2022b), indicating a dual approach to address both exhaustion and senescence. Other strategies focus on disrupting DNA damage signaling pathways that contribute to T cell senescence. Blockade of proteins involved in these pathways, such as ATM, ATR, or MAPK, has shown potential to prevent T cell senescence and enhance ICI efficacy in multiple cancer types in mice (Liu et al. 2022). Inhibiting DNA damage response pathways in conjunction with anti‐PD‐L1 significantly delayed tumor progression and improved survival in preclinical models (Chaib et al. 2024). Additionally, cytokine therapies, including IL‐2, IL‐7, IL‐12, and IL‐15, are being explored to rejuvenate T cells and boost anti‐tumor responses. For example, IL‐7 and IL‐15 have been shown to promote T cell survival, proliferation, and function, potentially reversing exhaustion and senescence (Kim et al. 2008). Recent clinical trials, such as a Phase I/II study combining pembrolizumab with IL‐12, have shown promising early results in enhancing T cell activation and reducing tumor burden in NSCLC, supporting the rationale for combining cytokines with ICIs to overcome T cell dysfunction (Kim et al. 2022). However, designing clinical trials for cancer patients with prior chemotherapy and radiotherapy is challenging due to the development of treatment resistance and the confounding effects of previous therapies on the immune system, complicating the evaluation of new treatments, especially senolytic agents.

### 8.2 Modulating the TME

Modulating the TME is a crucial strategy to enhance the efficacy of immunotherapy. The TME is characterized by an abundance of Tregs, TAMs, and MDSCs, all of which cause senescence and exhaustion (Chen et al. 2005; Togashi et al. 2019; Lasser et al. 2024). Depleting these immunosuppressive cell populations is a promising strategy to prevent T cell senescence and enhance immunotherapy. For example, CSF‐1R inhibitors have been shown to reduce TAMs, resulting in a less suppressive TME and enhanced T cell‐mediated anti‐tumor responses (Butowski et al. 2016). Additionally, anti‐CD25 therapies targeting Tregs have demonstrated efficacy in preclinical GBM models by decreasing Treg populations and augmenting cytotoxic T cell function (Solomon et al. 2020; Nishikawa and Sakaguchi 2014). Inhibiting the cytokines released by these cells is another potential strategy to improve T cell function and therapeutic outcomes (Hambardzumyan et al. 2016; Quail and Joyce 2017). For instance, TGF‐β inhibitors, such as galunisertib, have been demonstrated to reduce the immunosuppressive activity of the TME, thereby enhancing the effectiveness of ICIs and ACT (Holmgaard et al. 2018). Similarly, targeting IL‐10, a cytokine that suppresses T cell activation, is being explored as a method to reverse immunosuppression (Widodo et al. 2021). However, it remains to be determined whether the benefits of depleting immunosuppressive cells and inhibiting their cytokine secretion are mediated by preventing T cell senescence and exhaustion.

Metabolic reprogramming of T cells in the TME is an emerging approach to counteract the suppressive influence of the TME. Targeting metabolic pathways, such as mTOR, which regulates T cell differentiation and function, can help reprogram T cells to resist exhaustion and senescence. Rapamycin and other mTOR inhibitors have been shown to enhance T cell function and reduce senescence markers in preclinical studies (Waickman and Powell 2012; Chen et al. 2023b; Sullivan and Pearce 2015). Additionally, inhibitors of DNA damage response pathways, such as PARP inhibitors, may prevent T cell senescence by limiting DNA damage accumulation, thereby enhancing anti‐tumor activity (Li et al. 2023). Nutritional interventions, such as caloric restriction and antioxidant supplementation, are also being explored as non‐pharmacological approaches to modulate the TME and attenuate T cell senescence (Golonko et al. 2024; Yan et al. 2021). Caloric restriction has been shown to reduce inflammation and improve immune function, including T cell activity, in various cancer models (Golonko et al. 2024). For example, an antioxidant N‐acetylcysteine (NAC) mitigates oxidative stress, a key driver of senescence, and has shown potential in preclinical models to restore T cell function (Scheffel et al. 2018).

### 8.3 Enhancing T Cell Persistence and Function

Enhancing T cell persistence and functionality through genetic engineering and advanced cell modification techniques shows significant promise in overcoming T cell exhaustion and senescence in solid tumors. A key strategy involves engineering T cells with additional costimulatory domains, such as 4‐1BB or CD28, which enhance their activation, persistence, and resistance to exhaustion and senescence when utilized in CAR‐T cell therapies (López‐Cantillo et al. 2022; Poorebrahim et al. 2021). These modifications help sustain T cell effector functions, maintain cytokine production, and improve overall T cell survival in the challenging TME. Incorporating cytokine receptors into T cells has also shown the potential to bolster T cell persistence and function by promoting cell survival and reducing senescence‐associated markers (Bell and Gottschalk 2021). For example, IL‐15 transduction in CAR‐T cells enhances their expansion, persistence, and anti‐tumor activity, particularly in the immunosuppressive environment of solid tumors (Giuffrida et al. 2020). These modifications not only improve T cell survival but also help sustain anti‐tumor responses by mitigating the effects of immunosuppressive signals from the TME (Alizadeh et al. 2019). Additionally, engineering T cells to express anti‐senescent factors, such as telomerase or inhibitors of p16 and p21, targets intrinsic aging pathways, reducing senescence and boosting anti‐tumor activity (Janelle et al. 2021; Amor et al. 2020; Akincilar et al. 2016). Preclinical studies have demonstrated that T cells engineered to express telomerase maintain longer telomeres, exhibit better proliferative capacity, and show enhanced persistence in ACT settings (Rufer et al. 2001). Lastly, dual CAR‐T cell therapies, which target both tumor antigens and senescent cell markers may offer a novel approach to simultaneously address cancer and senescent cells within the TME. For instance, dual CAR‐T therapies targeting EGFRvIII on tumor cells alongside senescent cell markers like p16INK4a or Bcl‐2 may enhance anti‐tumor efficacy by reducing senescent cells that contribute to tumor growth and immunosuppression. This approach provides a comprehensive strategy by addressing both the direct elimination of cancer cells and the mitigation of the pro‐tumorigenic effects of senescent cells, potentially improving immunotherapy outcomes.

### 8.4 Senolytic Therapy

Several studies show that the accumulation of senescent cells in tumors is associated with decreased CD8+ T cell activation and proliferation (Amor et al. 2020; Toso et al. 2014; Eggert et al. 2016). Further, eliminating senescent cells in older individuals or those with TIS can improve the effectiveness of cancer immunotherapies (Amor et al. 2020; Kang et al. 2011). Senolytic therapies selectively target and induce apoptosis in senescent cells by disrupting their pro‐survival pathways, presenting a promising approach to mitigate their immunosuppressive effects in the TME. When combined with immunotherapies such as ICIs or ACT, senolytics have demonstrated synergistic effects in preclinical models of different tumors (Montero et al. 2011; Mustjoki et al. 2013; Salam et al. 2023). These combinations enhance cytotoxic T cell function, reduce tumor burden, and restore the immune landscape by reversing the suppressive profile of myeloid cells, thereby overcoming resistance to immunotherapy. For example, the senolytic agent ABT263 has been shown to improve the efficacy of anti‐PD‐L1 therapy by partially restoring the immunosuppressive phenotype of monocytes and enhancing CD8+ T cell activation and proliferation (Maggiorani et al. 2024; Saleh et al. 2020). Similarly, depletion of senescent cancer‐associated fibroblasts (CAFs) using the senolytic drug ABT‐199 (venetoclax) increases the proportion of activated CD8+ T cells, and its combination with immune checkpoint therapy significantly reduces tumor burden (Assouline et al. 2024). Furthermore, combining senolytic therapy to prevent tumor‐specific T cell senescence with anti‐PD‐L1 checkpoint blockade has also been found to synergistically enhance anti‐tumor immunity and improve immunotherapy outcomes in vivo (Liu et al. 2022).

Senolytics also show potential when combined with standard treatments such as TMZ and radiation therapy in preclinical models, suggesting their role in enhancing overall treatment efficacy (Fletcher‐Sananikone et al. 2021; Beltzig et al. 2022). By modulating the immune landscape, senolytics reverse the immunosuppressive profile of myeloid cells and restore cytotoxic T cell functions, ultimately reducing resistance to immunotherapy. Overall, targeting T cell exhaustion and senescence through a combination of immunomodulation, TME modulation, advanced cell engineering, and senolytic interventions provides a multifaceted approach to improving immunotherapy outcomes. However, further research is required to refine these strategies and determine their efficacy in clinical settings, paving the way for more effective and personalized cancer treatments.

## 9 Conclusions and Perspectives

The landscape of cancer immunotherapy has evolved significantly, yet challenges related to T cell exhaustion and senescence continue to impede progress. The persistent dysfunction of T cells within the TME underscores the need for novel strategies to enhance therapeutic efficacy. The evaluation of senolytics specifically for solid tumors remains in its nascent stages. Additionally, overcoming barriers to effective drug delivery, such as the dense extracellular matrix and abnormal vasculature typical of solid tumors, remains a significant hurdle for the administration of senolytics and other therapeutic agents. Advances in drug delivery technologies, including nanoparticle‐based systems and direct intratumoral administration, are being explored to enhance drug penetration and efficacy (Di Filippo et al. 2021). Further, recent findings suggest that cellular senescence, traditionally seen as a permanent state, might be more dynamic than previously believed and hence senescent cells may possess the potential for reversal and proliferation (Aguirre‐Ghiso 2007; Roberson et al. 2005). This insight opens exciting possibilities for developing therapies aimed at rejuvenating senescent T cells. Future research should prioritize the clinical trial of senolytics and T cell rejuvenation in combination with immunotherapies to address T cell senescence more effectively.

Key challenges include identifying reliable biomarkers for detecting senescent cells within the TME and distinguishing them from exhausted T cells. Identification of reliable biomarkers and innovative strategies to modulate the TME will be crucial. A multidisciplinary approach is essential to dissect senescence and exhaustion. Understanding the complex interactions between these two cell types and their interaction with other immune cells, such as B cells—which influence TCR diversity and senescence (Khan et al. 2023)—will be critical for developing next‐generation immunotherapies.

Moreover, reengineering the TME to overcome physical and biochemical barriers remains a critical challenge. Strategies such as metabolic reprogramming of immune cells (Pauken and Wherry 2015; Cerezo and Rocchi 2020) to enhance their resilience in hypoxic and nutrient‐depleted environments, remodeling stromal and extracellular matrix components (Mai et al. 2024; de Visser and Joyce 2023) to facilitate immune cell infiltration, and targeting suppressive cytokines secreted by senescent cells (Lelarge et al. 2024) hold significant promise. Further, Advanced CAR‐T designs to target cancer and senescent cells, resist exhaustion, or secrete immunomodulatory cytokines represent the next frontier in cellular therapies for solid tumors (Deng et al. 2024; Feucht and Abou‐El‐Enein 2020).

In summary, the future of cancer immunotherapy hinges on continued exploration and innovation. Addressing the challenges of T cells senescence and exhaustion, immune evasion, TME immunosuppression, and senescence‐immune cells interactions will be key for developing effective, transformative therapies. The path forward involves leveraging emerging insights and technologies to enhance treatment efficacy and improve patient outcomes. Future research must focus on understanding and overcoming barriers in the TME to unlock new potential treatments. Harnessing the mechanisms of senescence in the TME could lead to breakthroughs in enhancing the effectiveness of current treatments and discovering new therapeutic avenues.

## Author Contributions

T.D.A. and J.S.Y. drafted and edited the manuscript. T.D.A. drafted all the images.

## Conflicts of Interest

The authors declare no conflicts of interest.

## Data Availability

Data sharing is not applicable to this article as no new data were created or analyzed in this study.

## References

[acel70055-bib-0001] Acosta, J. C. , A. Banito , T. Wuestefeld , et al. 2013. “A Complex Secretory Program Orchestrated by the Inflammasome Controls Paracrine Senescence.” Nature Cell Biology 15: 978–990. 10.1038/ncb2784. 23770676 PMC3732483

[acel70055-bib-0002] Aghajani, M. , N. Jalilzadeh , A. Aghebati‐Maleki , et al. 2024. “Current Approaches in Glioblastoma Multiforme Immunotherapy.” Clinical & Translational Oncology 26: 1584–1612. 10.1007/s12094-024-03395-7. 38512448

[acel70055-bib-0003] Aguirre‐Ghiso, J. A. 2007. “Models, Mechanisms and Clinical Evidence for Cancer Dormancy.” Nature Reviews. Cancer 7: 834–846. 10.1038/nrc2256. 17957189 PMC2519109

[acel70055-bib-0004] Akbar, A. N. , and M. Vukmanovic‐Stejic . 2007. “Telomerase in T Lymphocytes: Use It and Lose It?” Journal of Immunology 178: 6689–6694. 10.4049/jimmunol.178.11.6689. 17513711

[acel70055-bib-0005] Akhavan, D. , D. Alizadeh , D. Wang , M. R. Weist , J. K. Shepphird , and C. E. Brown . 2019. “CAR T Cells for Brain Tumors: Lessons Learned and Road Ahead.” Immunological Reviews 290: 60–84. 10.1111/imr.12773. 31355493 PMC6771592

[acel70055-bib-0006] Akincilar, S. C. , B. Unal , and V. Tergaonkar . 2016. “Reactivation of Telomerase in Cancer.” Cellular and Molecular Life Sciences 73: 1659–1670. 10.1007/s00018-016-2146-9. 26846696 PMC4805692

[acel70055-bib-0007] Alizadeh, D. , R. A. Wong , X. Yang , et al. 2019. “IL15 Enhances CAR‐T Cell Antitumor Activity by Reducing mTORC1 Activity and Preserving Their Stem Cell Memory Phenotype.” Cancer Immunology Research 7: 759–772. 10.1158/2326-6066.Cir-18-0466. 30890531 PMC6687561

[acel70055-bib-0008] Amor, C. , J. Feucht , J. Leibold , et al. 2020. “Senolytic CAR T Cells Reverse Senescence‐Associated Pathologies.” Nature 583: 127–132. 10.1038/s41586-020-2403-9. 32555459 PMC7583560

[acel70055-bib-0009] Anderson, A. C. , N. Joller , and V. K. Kuchroo . 2016. “Lag‐3, Tim‐3, and TIGIT: Co‐Inhibitory Receptors With Specialized Functions in Immune Regulation.” Immunity 44: 989–1004. 10.1016/j.immuni.2016.05.001. 27192565 PMC4942846

[acel70055-bib-0010] Andtbacka, R. H. , R. H. I. Andtbacka , H. L. Kaufman , et al. 2015. “Talimogene Laherparepvec Improves Durable Response Rate in Patients With Advanced Melanoma.” Journal of Clinical Oncology 33, no. 25: 2780–2788. 10.1200/jco.2014.58.3377. 26014293

[acel70055-bib-0011] Antonios, J. P. , H. Soto , R. G. Everson , et al. 2016. “PD‐1 Blockade Enhances the Vaccination‐Induced Immune Response in Glioma.” JCI Insight 1: 87059. 10.1172/jci.insight.87059. PMC4951098 27453950

[acel70055-bib-0012] Asija, S. , A. Chatterjee , J. S. Goda , S. Yadav , G. Chekuri , and R. Purwar . 2023. “Oncolytic Immunovirotherapy for High‐Grade Gliomas: A Novel and an Evolving Therapeutic Option.” Frontiers in Immunology 14: 1118246. 10.3389/fimmu.2023.1118246. 37006286 PMC10050572

[acel70055-bib-0013] Assouline, B. , R. Kahn , L. Hodali , et al. 2024. “Senescent Cancer‐Associated Fibroblasts in Pancreatic Adenocarcinoma Restrict CD8(+) T Cell Activation and Limit Responsiveness to Immunotherapy in Mice.” Nature Communications 15: 6162. 10.1038/s41467-024-50441-7. PMC11263607 39039076

[acel70055-bib-0014] Bagley, S. J. , M. Logun , J. A. Fraietta , et al. 2024. “Intrathecal Bivalent CAR T Cells Targeting EGFR and IL13Rα2 in Recurrent Glioblastoma: Phase 1 Trial Interim Results.” Nature Medicine 30: 1320–1329. 10.1038/s41591-024-02893-z. PMC13123313 38480922

[acel70055-bib-0015] Bausart, M. , V. Préat , and A. Malfanti . 2022. “Immunotherapy for Glioblastoma: The Promise of Combination Strategies.” Journal of Experimental & Clinical Cancer Research 41: 35. 10.1186/s13046-022-02251-2. 35078492 PMC8787896

[acel70055-bib-0016] Bell, M. , and S. Gottschalk . 2021. “Engineered Cytokine Signaling to Improve CAR T Cell Effector Function.” Frontiers in Immunology 12: 684642. 10.3389/fimmu.2021.684642. 34177932 PMC8220823

[acel70055-bib-0017] Beltzig, L. , M. Christmann , and B. Kaina . 2022. “Abrogation of Cellular Senescence Induced by Temozolomide in Glioblastoma Cells: Search for Senolytics.” Cells 11: 2588. 10.3390/cells11162588. 36010664 PMC9406955

[acel70055-bib-0018] Brown, C. E. , J. C. Hibbard , D. Alizadeh , et al. 2024. “Locoregional Delivery of IL‐13Rα2‐Targeting CAR‐T Cells in Recurrent High‐Grade Glioma: A Phase 1 Trial.” Nature Medicine 30: 1001–1012. 10.1038/s41591-024-02875-1. PMC11031404 38454126

[acel70055-bib-0019] Butowski, N. , H. Colman , J. F. de Groot , et al. 2016. “Orally Administered Colony Stimulating Factor 1 Receptor Inhibitor PLX3397 in Recurrent Glioblastoma: An ivy Foundation Early Phase Clinical Trials Consortium Phase II Study.” Neuro‐Oncology 18: 557–564. 10.1093/neuonc/nov245. 26449250 PMC4799682

[acel70055-bib-0020] Callender, L. A. , E. C. Carroll , R. W. J. Beal , et al. 2018. “Human CD8(+) EMRA T Cells Display a Senescence‐Associated Secretory Phenotype Regulated by p38 MAPK.” Aging Cell 17: 12675. 10.1111/acel.12675. PMC5770853 29024417

[acel70055-bib-0021] Campisi, J. 2013. “Aging, Cellular Senescence, and Cancer.” Annual Review of Physiology 75: 685–705. 10.1146/annurev-physiol-030212-183653. PMC4166529 23140366

[acel70055-bib-0022] Cao, J. , S. Liao , F. Zeng , Q. Liao , G. Luo , and Y. Zhou . 2023. “Effects of Altered Glycolysis Levels on CD8(+) T Cell Activation and Function.” Cell Death & Disease 14: 407. 10.1038/s41419-023-05937-3. 37422501 PMC10329707

[acel70055-bib-0023] Cerezo, M. , and S. Rocchi . 2020. “Cancer Cell Metabolic Reprogramming: A Keystone for the Response to Immunotherapy.” Cell Death & Disease 11: 964. 10.1038/s41419-020-03175-5. 33177494 PMC7658964

[acel70055-bib-0024] Chaib, S. , J. A. López‐Domínguez , M. Lalinde‐Gutiérrez , et al. 2024. “The Efficacy of Chemotherapy Is Limited by Intratumoral Senescent Cells Expressing PD‐L2.” Nature Cancer 5: 448–462. 10.1038/s43018-023-00712-x. 38267628 PMC10965441

[acel70055-bib-0025] Chaib, S. , J. A. López‐Domínguez , M. Lalinde‐Gutiérrez , et al. 2022. “The Efficacy of Chemotherapy Is Limited by Intratumoural Senescent Cells That Persist Through the Upregulation of PD‐L2.” bioRxiv 20: 1681. 10.1101/2022.11.04.501681.

[acel70055-bib-0026] Chen, A. C. Y. , S. Jaiswal , D. Martinez , et al. 2024. “The Aged Tumor Microenvironment Limits T Cell Control of Cancer.” Nature Immunology 25: 1033–1045. 10.1038/s41590-024-01828-7. 38745085 PMC11500459

[acel70055-bib-0027] Chen, D. S. , and I. Mellman . 2013. “Oncology Meets Immunology: The Cancer‐Immunity Cycle.” Immunity 39: 1–10. 10.1016/j.immuni.2013.07.012. 23890059

[acel70055-bib-0028] Chen, D. S. , and I. Mellman . 2017. “Elements of Cancer Immunity and the Cancer–Immune Set Point.” Nature 541: 321–330. 10.1038/nature21349. 28102259

[acel70055-bib-0029] Chen, H. A. , Y. J. Ho , R. Mezzadra , et al. 2023a. “Senescence Rewires Microenvironment Sensing to Facilitate Antitumor Immunity.” Cancer Discovery 13: 432–453. 10.1158/2159-8290.Cd-22-0528. 36302222 PMC9901536

[acel70055-bib-0030] Chen, J. J. , J. J. W. Chen , Y.‐C. Lin , et al. 2005. “Tumor‐Associated Macrophages: The Double‐Edged Sword in Cancer Progression.” Journal of Clinical Oncology 23, no. 5: 953–964. 10.1200/jco.2005.12.172. 15598976

[acel70055-bib-0031] Chen, Y. , Z. Xu , H. Sun , et al. 2023b. “Regulation of CD8+ T Memory and Exhaustion by the mTOR Signals.” Cellular & Molecular Immunology 20: 1023–1039. 10.1038/s41423-023-01064-3. 37582972 PMC10468538

[acel70055-bib-0032] Chow, A. , K. Perica , C. A. Klebanoff , and J. D. Wolchok . 2022. “Clinical Implications of T Cell Exhaustion for Cancer Immunotherapy.” Nature Reviews. Clinical Oncology 19: 775–790. 10.1038/s41571-022-00689-z. PMC10984554 36216928

[acel70055-bib-0033] Coppé, J. P. , P. Y. Desprez , A. Krtolica , and J. Campisi . 2010. “The Senescence‐Associated Secretory Phenotype: The Dark Side of Tumor Suppression.” Annual Review of Pathology 5: 99–118. 10.1146/annurev-pathol-121808-102144. PMC4166495 20078217

[acel70055-bib-0034] Coppé, J. P. , C. K. Patil , F. Rodier , et al. 2008. “Senescence‐Associated Secretory Phenotypes Reveal Cell‐Nonautonomous Functions of Oncogenic RAS and the p53 Tumor Suppressor.” PLoS Biology 6: 2853–2868. 10.1371/journal.pbio.0060301. 19053174 PMC2592359

[acel70055-bib-0035] Covre, L. P. , R. P. H. De Maeyer , D. C. O. Gomes , and A. N. Akbar . 2020. “The Role of Senescent T Cells in Immunopathology.” Aging Cell 19: e13272. 10.1111/acel.13272. 33166035 PMC7744956

[acel70055-bib-0036] de Visser, K. E. , and J. A. Joyce . 2023. “The Evolving Tumor Microenvironment: From Cancer Initiation to Metastatic Outgrowth.” Cancer Cell 41: 374–403. 10.1016/j.ccell.2023.02.016. 36917948

[acel70055-bib-0037] Dehne, N. , J. Mora , D. Namgaladze , A. Weigert , and B. Brüne . 2017. “Cancer Cell and Macrophage Cross‐Talk in the Tumor Microenvironment.” Current Opinion in Pharmacology 35: 12–19. 10.1016/j.coph.2017.04.007. 28538141

[acel70055-bib-0038] Demaria, M. , M. N. O'Leary , J. Chang , et al. 2017. “Cellular Senescence Promotes Adverse Effects of Chemotherapy and Cancer Relapse.” Cancer Discovery 7: 165–176. 10.1158/2159-8290.Cd-16-0241. 27979832 PMC5296251

[acel70055-bib-0039] Deng, Y. , A. Kumar , K. Xie , et al. 2024. “Targeting Senescent Cells With NKG2D‐CAR T Cells.” Cell Death Discovery 10: 217. 10.1038/s41420-024-01976-7. 38704364 PMC11069534

[acel70055-bib-0040] Desjardins, A. , M. Gromeier , J. E. Herndon II , et al. 2018. “Recurrent Glioblastoma Treated With Recombinant Poliovirus.” New England Journal of Medicine 379: 150–161. 10.1056/NEJMoa1716435. 29943666 PMC6065102

[acel70055-bib-0041] Di Filippo, L. D. , J. L. Duarte , M. T. Luiz , J. T. C. de Araújo , and M. Chorilli . 2021. “Drug Delivery Nanosystems in Glioblastoma Multiforme Treatment: Current State of the Art.” Current Neuropharmacology 19: 787–812. 10.2174/1570159x18666200831160627. 32867643 PMC8686306

[acel70055-bib-0042] Dudley, M. E. , and S. A. Rosenberg . 2003. “Adoptive‐Cell‐Transfer Therapy for the Treatment of Patients With Cancer.” Nature Reviews. Cancer 3: 666–675. 10.1038/nrc1167. 12951585 PMC2305722

[acel70055-bib-0043] Durgin, J. S. , F. Henderson Jr. , M. L. P. Nasrallah , et al. 2021. “Case Report: Prolonged Survival Following EGFRvIII CAR T Cell Treatment for Recurrent Glioblastoma.” Frontiers in Oncology 11: 669071. 10.3389/fonc.2021.669071. 34026647 PMC8138201

[acel70055-bib-0044] Eggert, T. , K. Wolter , J. Ji , et al. 2016. “Distinct Functions of Senescence‐Associated Immune Responses in Liver Tumor Surveillance and Tumor Progression.” Cancer Cell 30: 533–547. 10.1016/j.ccell.2016.09.003. 27728804 PMC7789819

[acel70055-bib-0045] Everson, R. G. , J. P. Antonios , D. N. Lisiero , et al. 2016. “Efficacy of Systemic Adoptive Transfer Immunotherapy Targeting NY‐ESO‐1 for Glioblastoma.” Neuro‐Oncology 18: 368–378. 10.1093/neuonc/nov153. 26330563 PMC4767237

[acel70055-bib-0046] Everson, R. G. , W. Hugo , L. Sun , et al. 2024. “TLR Agonists Polarize Interferon Responses in Conjunction With Dendritic Cell Vaccination in Malignant Glioma: A Randomized Phase II Trial.” Nature Communications 15: 3882. 10.1038/s41467-024-48073-y. PMC11078958 38719809

[acel70055-bib-0047] Faget, D. V. , Q. Ren , and S. A. Stewart . 2019. “Unmasking Senescence: Context‐Dependent Effects of SASP in Cancer.” Nature Reviews. Cancer 19: 439–453. 10.1038/s41568-019-0156-2. 31235879

[acel70055-bib-0048] Fane, M. , and A. T. Weeraratna . 2020. “How the Ageing Microenvironment Influences Tumour Progression.” Nature Reviews. Cancer 20: 89–106. 10.1038/s41568-019-0222-9. 31836838 PMC7377404

[acel70055-bib-0049] Fenstermaker, R. A. , M. J. Ciesielski , J. Qiu , et al. 2016. “Clinical Study of a Survivin Long Peptide Vaccine (SurVaxM) in Patients With Recurrent Malignant Glioma.” Cancer Immunology, Immunotherapy 65: 1339–1352. 10.1007/s00262-016-1890-x. 27576783 PMC5069322

[acel70055-bib-0050] Fernandes, J. R. , T. N. C. Pinto , L. B. Arruda , et al. 2022. “Age‐Associated Phenotypic Imbalance in TCD4 and TCD8 Cell Subsets: Comparison Between Healthy Aged, Smokers, COPD Patients and Young Adults.” Immunity & Ageing 19: 9. 10.1186/s12979-022-00267-y. 35164774 PMC8842531

[acel70055-bib-0051] Feucht, J. , and M. Abou‐El‐Enein . 2020. “Senolytic CAR T Cells in Solid Tumors and Age‐Related Pathologies.” Molecular Therapy 28: 2108–2110. 10.1016/j.ymthe.2020.08.011. 32841587 PMC7545000

[acel70055-bib-0052] Fletcher‐Sananikone, E. , S. Kanji , N. Tomimatsu , et al. 2021. “Elimination of Radiation‐Induced Senescence in the Brain Tumor Microenvironment Attenuates Glioblastoma Recurrence.” Cancer Research 81: 5935–5947. 10.1158/0008-5472.Can-21-0752. 34580063 PMC9724593

[acel70055-bib-0053] Fox, C. J. , P. S. Hammerman , and C. B. Thompson . 2005. “Fuel Feeds Function: Energy Metabolism and the T‐Cell Response.” Nature Reviews. Immunology 5: 844–852. 10.1038/nri1710. 16239903

[acel70055-bib-0054] Fu, Z. , H. Xu , L. Yue , et al. 2023. “Immunosenescence and Cancer: Opportunities and Challenges.” Medicine 102, no. 47: e36045. 10.1097/md.0000000000036045. 38013358 PMC10681516

[acel70055-bib-0055] Gabrilovich, D. I. , and S. Nagaraj . 2009. “Myeloid‐Derived Suppressor Cells as Regulators of the Immune System.” Nature Reviews Immunology 9: 162–174. 10.1038/nri2506. PMC2828349 19197294

[acel70055-bib-0056] Gajewski, T. F. , H. Schreiber , and Y. X. Fu . 2013. “Innate and Adaptive Immune Cells in the Tumor Microenvironment.” Nature Immunology 14: 1014–1022. 10.1038/ni.2703. 24048123 PMC4118725

[acel70055-bib-0057] Gasek, N. S. , G. A. Kuchel , J. L. Kirkland , and M. Xu . 2021. “Strategies for Targeting Senescent Cells in Human Disease.” Nature Aging 1: 870–879. 10.1038/s43587-021-00121-8. 34841261 PMC8612694

[acel70055-bib-0058] Gatto, L. , V. Di Nunno , A. Tosoni , S. Bartolini , L. Ranieri , and E. Franceschi . 2023. “DCVax‐L Vaccination in Patients With Glioblastoma: Real Promise or Negative Trial? The Debate Is Open.” Cancers 15, no. 12: 3251. 10.3390/cancers15123251. 37370860 PMC10296384

[acel70055-bib-0059] Gilioli, D. , S. Fusco , T. Tavella , et al. 2022. “Therapy‐Induced Senescence Upregulates Antigen Presentation Machinery and Triggers Anti‐Tumor Immunity in Acute Myeloid Leukemia.” bioRxiv 20: 658. 10.1101/2022.11.17.515658.

[acel70055-bib-0060] Giuffrida, L. , K. Sek , M. A. Henderson , et al. 2020. “IL‐15 Preconditioning Augments CAR T Cell Responses to Checkpoint Blockade for Improved Treatment of Solid Tumors.” Molecular Therapy 28: 2379–2393. 10.1016/j.ymthe.2020.07.018. 32735774 PMC7647667

[acel70055-bib-0061] Golonko, A. , T. Pienkowski , R. Swislocka , et al. 2024. “Dietary Factors and Their Influence on Immunotherapy Strategies in Oncology: A Comprehensive Review.” Cell Death & Disease 15: 254. 10.1038/s41419-024-06641-6. 38594256 PMC11004013

[acel70055-bib-0062] Gonzalez‐Meljem, J. M. , J. R. Apps , H. C. Fraser , and J. P. Martinez‐Barbera . 2018. “Paracrine Roles of Cellular Senescence in Promoting Tumourigenesis.” British Journal of Cancer 118: 1283–1288. 10.1038/s41416-018-0066-1. 29670296 PMC5959857

[acel70055-bib-0063] Grimmett, E. , B. al‐Share , M. B. Alkassab , et al. 2022. “Cancer Vaccines: Past, Present and Future; a Review Article.” Discover Oncology 13: 31. 10.1007/s12672-022-00491-4. 35576080 PMC9108694

[acel70055-bib-0064] Hambardzumyan, D. , D. H. Gutmann , and H. Kettenmann . 2016. “The Role of Microglia and Macrophages in Glioma Maintenance and Progression.” Nature Neuroscience 19: 20–27. 10.1038/nn.4185. 26713745 PMC4876023

[acel70055-bib-0065] Hamilton, S. E. , and S. C. Jameson . 2012. “CD8 T Cell Quiescence Revisited.” Trends in Immunology 33: 224–230. 10.1016/j.it.2012.01.007. 22361353 PMC3348359

[acel70055-bib-0066] Han, S. , P. Georgiev , A. E. Ringel , A. H. Sharpe , and M. C. Haigis . 2023. “Age‐Associated Remodeling of T Cell Immunity and Metabolism.” Cell Metabolism 35: 36–55. 10.1016/j.cmet.2022.11.005. 36473467 PMC10799654

[acel70055-bib-0067] He, S. , and N. E. Sharpless . 2017. “Senescence in Health and Disease.” Cell 169: 1000–1011. 10.1016/j.cell.2017.05.015. 28575665 PMC5643029

[acel70055-bib-0068] Henson, S. M. , R. Macaulay , N. E. Riddell , C. J. Nunn , and A. N. Akbar . 2015. “Blockade of PD‐1 or p38 MAP Kinase Signaling Enhances Senescent Human CD8(+) T‐Cell Proliferation by Distinct Pathways.” European Journal of Immunology 45: 1441–1451. 10.1002/eji.201445312. 25707450

[acel70055-bib-0069] Hernandez‐Segura, A. , J. Nehme , and M. Demaria . 2018. “Hallmarks of Cellular Senescence.” Trends in Cell Biology 28: 436–453. 10.1016/j.tcb.2018.02.001. 29477613

[acel70055-bib-0070] Hodes, R. J. , K. S. Hathcock , and N. P. Weng . 2002. “Telomeres in T and B Cells.” Nature Reviews. Immunology 2: 699–706. 10.1038/nri890. 12209138

[acel70055-bib-0071] Hodi, F. S. , S. J. O'Day , D. F. McDermott , et al. 2010. “Improved Survival With Ipilimumab in Patients With Metastatic Melanoma.” New England Journal of Medicine 363: 711–723. 10.1056/NEJMoa1003466. 20525992 PMC3549297

[acel70055-bib-0072] Holmgaard, R. B. , D. A. Schaer , Y. Li , et al. 2018. “Targeting the TGFβ Pathway With Galunisertib, a TGFβRI Small Molecule Inhibitor, Promotes Anti‐Tumor Immunity Leading to Durable, Complete Responses, as Monotherapy and in Combination With Checkpoint Blockade.” Journal for Immunotherapy of Cancer 6: 47. 10.1186/s40425-018-0356-4. 29866156 PMC5987416

[acel70055-bib-0073] Hou, J. , K. X. Chen , C. He , et al. 2024. “Aged Bone Marrow Macrophages Drive Systemic Aging and Age‐Related Dysfunction via Extracellular Vesicle‐Mediated Induction of Paracrine Senescence.” Nature Aging 4: 1562–1581. 10.1038/s43587-024-00694-0. 39266768 PMC11564114

[acel70055-bib-0074] Hu, L. , H. Li , M. Zi , et al. 2022. “Why Senescent Cells Are Resistant to Apoptosis: An Insight for Senolytic Development.” Frontiers in Cell and Development Biology 10: 822816. 10.3389/fcell.2022.822816. PMC8890612 35252191

[acel70055-bib-0075] Huang, M. , Y. Wang , L. Fang , et al. 2024. “T Cell Senescence: A New Perspective on Immunotherapy in Lung Cancer.” Frontiers in Immunology 15: 1338680. 10.3389/fimmu.2024.1338680. 38415245 PMC10896971

[acel70055-bib-0076] Janelle, V. , M. Neault , M. È. Lebel , et al. 2021. “p16(INK4a) Regulates Cellular Senescence in PD‐1‐Expressing Human T Cells.” Frontiers in Immunology 12: 698565. 10.3389/fimmu.2021.698565. 34434190 PMC8381277

[acel70055-bib-0077] Jiang, Y. , Y. Li , and B. Zhu . 2015. “T‐Cell Exhaustion in the Tumor Microenvironment.” Cell Death & Disease 6: e1792. 10.1038/cddis.2015.162. 26086965 PMC4669840

[acel70055-bib-0078] Jing, X. , F. Yang , C. Shao , et al. 2019. “Role of Hypoxia in Cancer Therapy by Regulating the Tumor Microenvironment.” Molecular Cancer 18: 157. 10.1186/s12943-019-1089-9. 31711497 PMC6844052

[acel70055-bib-0079] Kang, T.‐W. , T. Yevsa , N. Woller , et al. 2011. “Senescence Surveillance of Pre‐Malignant Hepatocytes Limits Liver Cancer Development.” Nature 479: 547–551. 10.1038/nature10599. 22080947

[acel70055-bib-0080] Kasakovski, D. , L. Xu , and Y. Li . 2018. “T Cell Senescence and CAR‐T Cell Exhaustion in Hematological Malignancies.” Journal of Hematology & Oncology 11: 91. 10.1186/s13045-018-0629-x. 29973238 PMC6032767

[acel70055-bib-0081] Katsikis, P. D. , K. J. Ishii , and C. Schliehe . 2024. “Challenges in Developing Personalized Neoantigen Cancer Vaccines.” Nature Reviews. Immunology 24: 213–227. 10.1038/s41577-023-00937-y. PMC12001822 37783860

[acel70055-bib-0082] Kaufman, H. L. , F. J. Kohlhapp , and A. Zloza . 2015. “Oncolytic Viruses: A New Class of Immunotherapy Drugs.” Nature Reviews. Drug Discovery 14: 642–662. 10.1038/nrd4663. 26323545 PMC7097180

[acel70055-bib-0083] Kazemi, M. H. , M. Sadri , A. Najafi , et al. 2022. “Tumor‐Infiltrating Lymphocytes for Treatment of Solid Tumors: It Takes Two to Tango?” Frontiers in Immunology 13: 1018962. 10.3389/fimmu.2022.1018962. 36389779 PMC9651159

[acel70055-bib-0084] Khan, S. , M. Chakraborty , F. Wu , et al. 2023. “B Cells Promote T Cell Immunosenescence and Mammalian Aging Parameters.” bioRxiv 20: 363. 10.1101/2023.09.12.556363.

[acel70055-bib-0085] Kim, E. J. , Y. H. Cho , D. H. Kim , et al. 2022. “A Phase I/IIa Randomized Trial Evaluating the Safety and Efficacy of SNK01 Plus Pembrolizumab in Patients With Stage IV Non‐Small Cell Lung Cancer.” Cancer Research and Treatment 54: 1005–1016. 10.4143/crt.2021.986. 34856706 PMC9582480

[acel70055-bib-0086] Kim, H. R. , K. A. Hwang , S. H. Park , and I. Kang . 2008. “IL‐7 and IL‐15: Biology and Roles in T‐Cell Immunity in Health and Disease.” Critical Reviews in Immunology 28: 325–339. 10.1615/critrevimmunol.v28.i4.40. 19166383

[acel70055-bib-0087] Klebanoff, C. A. , S. S. Chandran , B. M. Baker , S. A. Quezada , and A. Ribas . 2023. “T Cell Receptor Therapeutics: Immunological Targeting of the Intracellular Cancer Proteome.” Nature Reviews. Drug Discovery 22: 996–1017. 10.1038/s41573-023-00809-z. 37891435 PMC10947610

[acel70055-bib-0088] Kousa, A. I. , L. Jahn , K. Zhao , et al. 2024. “Age‐Related Epithelial Defects Limit Thymic Function and Regeneration.” Nature Immunology 25: 1593–1606. 10.1038/s41590-024-01915-9. 39112630 PMC11362016

[acel70055-bib-0089] Kunert, A. , E. A. Basak , D. P. Hurkmans , et al. 2019. “CD45RA(+)CCR7(−) CD8 T Cells Lacking Co‐Stimulatory Receptors Demonstrate Enhanced Frequency in Peripheral Blood of NSCLC Patients Responding to Nivolumab.” Journal for Immunotherapy of Cancer 7: 149. 10.1186/s40425-019-0608-y. 31176366 PMC6555948

[acel70055-bib-0090] Lanna, A. , B. Vaz , C. D'Ambra , et al. 2022. “An Intercellular Transfer of Telomeres Rescues T Cells From Senescence and Promotes Long‐Term Immunological Memory.” Nature Cell Biology 24: 1461–1474. 10.1038/s41556-022-00991-z. 36109671 PMC7613731

[acel70055-bib-0091] Lasry, A. , and Y. Ben‐Neriah . 2015. “Senescence‐Associated Inflammatory Responses: Aging and Cancer Perspectives.” Trends in Immunology 36: 217–228. 10.1016/j.it.2015.02.009. 25801910

[acel70055-bib-0092] Lasser, S. A. , F. G. Ozbay Kurt , I. Arkhypov , J. Utikal , and V. Umansky . 2024. “Myeloid‐Derived Suppressor Cells in Cancer and Cancer Therapy.” Nature Reviews. Clinical Oncology 21: 147–164. 10.1038/s41571-023-00846-y. 38191922

[acel70055-bib-0093] Lelarge, V. , R. Capelle , F. Oger , T. Mathieu , and B. Le Calvé . 2024. “Senolytics: From Pharmacological Inhibitors to Immunotherapies, a Promising Future for Patients' Treatment.” Npj Aging 10, no. 1: 12. 10.1038/s41514-024-00138-4. 38321020 PMC10847408

[acel70055-bib-0094] Li, Q. , W. Qian , Y. Zhang , L. Hu , S. Chen , and Y. Xia . 2023. “A New Wave of Innovations Within the DNA Damage Response.” Signal Transduction and Targeted Therapy 8: 338. 10.1038/s41392-023-01548-8. 37679326 PMC10485079

[acel70055-bib-0095] Lian, J. , Y. Yue , W. Yu , and Y. Zhang . 2020. “Immunosenescence: A Key Player in Cancer Development.” Journal of Hematology & Oncology 13: 151. 10.1186/s13045-020-00986-z. 33168037 PMC7653700

[acel70055-bib-0096] Liang, Z. , X. Dong , Z. Zhang , Q. Zhang , and Y. Zhao . 2022. “Age‐Related Thymic Involution: Mechanisms and Functional Impact.” Aging Cell 21: e13671. 10.1111/acel.13671. 35822239 PMC9381902

[acel70055-bib-0097] Liau, L. M. , K. Ashkan , S. Brem , et al. 2023. “Association of Autologous Tumor Lysate‐Loaded Dendritic Cell Vaccination With Extension of Survival Among Patients With Newly Diagnosed and Recurrent Glioblastoma: A Phase 3 Prospective Externally Controlled Cohort Trial.” JAMA Oncology 9: 112–121. 10.1001/jamaoncol.2022.5370. 36394838 PMC9673026

[acel70055-bib-0098] Lin, M. J. , J. Svensson‐Arvelund , G. S. Lubitz , et al. 2022. “Cancer Vaccines: The Next Immunotherapy Frontier.” Nature Cancer 3: 911–926. 10.1038/s43018-022-00418-6. 35999309

[acel70055-bib-0099] Liu, W. , P. Stachura , H. C. Xu , et al. 2020a. “Senescent Tumor CD8(+) T Cells: Mechanisms of Induction and Challenges to Immunotherapy.” Cancers (Basel) 12: 2828. 10.3390/cancers12102828. 33008037 PMC7601312

[acel70055-bib-0100] Liu, X. , D. F. Hoft , and G. Peng . 2020b. “Senescent T Cells Within Suppressive Tumor Microenvironments: Emerging Target for Tumor Immunotherapy.” Journal of Clinical Investigation 130: 1073–1083. 10.1172/jci133679. 32118585 PMC7269563

[acel70055-bib-0101] Liu, X. , W. Mo , J. Ye , et al. 2018. “Regulatory T Cells Trigger Effector T Cell DNA Damage and Senescence Caused by Metabolic Competition.” Nature Communications 9: 249. 10.1038/s41467-017-02689-5. PMC5770447 29339767

[acel70055-bib-0102] Liu, X. , F. Si , D. Bagley , et al. 2022. “Blockades of Effector T Cell Senescence and Exhaustion Synergistically Enhance Antitumor Immunity and Immunotherapy.” Journal for Immunotherapy of Cancer 10: e005020. 10.1136/jitc-2022-005020. 36192086 PMC9535198

[acel70055-bib-0103] Liu, Z. , Q. Liang , Y. Ren , et al. 2023. “Immunosenescence: Molecular Mechanisms and Diseases.” Signal Transduction and Targeted Therapy 8: 200. 10.1038/s41392-023-01451-2. 37179335 PMC10182360

[acel70055-bib-0104] Liu, Z. , Q. Meng , J. Bartek Jr. , et al. 2017. “Tumor‐Infiltrating Lymphocytes (TILs) From Patients With Glioma.” Oncoimmunology 6: e1252894. 10.1080/2162402x.2016.1252894. 28344863 PMC5353900

[acel70055-bib-0105] López‐Cantillo, G. , C. Urueña , B. A. Camacho , and C. Ramírez‐Segura . 2022. “CAR‐T Cell Performance: How to Improve Their Persistence?” Frontiers in Immunology 13: 878209. 10.3389/fimmu.2022.878209. 35572525 PMC9097681

[acel70055-bib-0106] Lu, C. , and Y. Tan . 2024. “Promising Immunotherapy Targets: TIM3, LAG3, and TIGIT Joined the Party.” Molecular Therapy – Oncolytics 32: 200773. 10.1016/j.omton.2024.200773. PMC10905042 38596295

[acel70055-bib-0107] Maggiorani, D. , O. le , V. Lisi , et al. 2024. “Senescence Drives Immunotherapy Resistance by Inducing an Immunosuppressive Tumor Microenvironment.” Nature Communications 15: 2435. 10.1038/s41467-024-46769-9. PMC10948808 38499573

[acel70055-bib-0108] Mai, Z. , Y. Lin , P. Lin , X. Zhao , and L. Cui . 2024. “Modulating Extracellular Matrix Stiffness: A Strategic Approach to Boost Cancer Immunotherapy.” Cell Death & Disease 15: 307. 10.1038/s41419-024-06697-4. 38693104 PMC11063215

[acel70055-bib-0109] Maity, A. , R. Mick , A. C. Huang , et al. 2018. “A Phase I Trial of Pembrolizumab With Hypofractionated Radiotherapy in Patients With Metastatic Solid Tumours.” British Journal of Cancer 119: 1200–1207. 10.1038/s41416-018-0281-9. 30318516 PMC6251028

[acel70055-bib-0110] Marin, I. , O. Boix , A. Garcia‐Garijo , et al. 2023. “Cellular Senescence Is Immunogenic and Promotes Antitumor Immunity.” Cancer Discovery 13: 410–431. 10.1158/2159-8290.Cd-22-0523. 36302218 PMC7614152

[acel70055-bib-0111] Martin, M. D. , and V. P. Badovinac . 2018. “Defining Memory CD8 T Cell.” Frontiers in Immunology 9: 2692. 10.3389/fimmu.2018.02692. 30515169 PMC6255921

[acel70055-bib-0112] Mejia‐Ramirez, E. , and M. C. Florian . 2020. “Understanding Intrinsic Hematopoietic Stem Cell Aging.” Haematologica 105: 22–37. 10.3324/haematol.2018.211342. 31806687 PMC6939535

[acel70055-bib-0113] Mellman, I. , D. S. Chen , T. Powles , and S. J. Turley . 2023. “The Cancer‐Immunity Cycle: Indication, Genotype, and Immunotype.” Immunity 56: 2188–2205. 10.1016/j.immuni.2023.09.011. 37820582

[acel70055-bib-0114] Mellman, I. , G. Coukos , and G. Dranoff . 2011. “Cancer Immunotherapy Comes of Age.” Nature 480: 480–489. 10.1038/nature10673. 22193102 PMC3967235

[acel70055-bib-0115] Mhaidly, R. , and F. Mechta‐Grigoriou . 2021. “Role of Cancer‐Associated Fibroblast Subpopulations in Immune Infiltration, as a New Means of Treatment in Cancer.” Immunological Reviews 302: 259–272. 10.1111/imr.12978. 34013544 PMC8360036

[acel70055-bib-0116] Migliorini, D. , and V. Dutoit . 2016. “ATIM‐21. IMA950 Peptide‐Based Vaccine Adjuvanted With Poly‐ICLC in Combination With Standard Therapy in Newly Diagnosed HLA‐A2 Glioblastoma Patients: Preliminary Results.” Neuro‐Oncology 18: vi22. 10.1093/neuonc/now212.086.

[acel70055-bib-0117] Migliorini, D. , V. Dutoit , M. Allard , et al. 2019. “Phase I/II Trial Testing Safety and Immunogenicity of the Multipeptide IMA950/Poly‐ICLC Vaccine in Newly Diagnosed Adult Malignant Astrocytoma Patients.” Neuro‐Oncology 21: 923–933. 10.1093/neuonc/noz040. 30753611 PMC6620642

[acel70055-bib-0118] Milanovic, M. , D. N. Y. Fan , D. Belenki , et al. 2018. “Senescence‐Associated Reprogramming Promotes Cancer Stemness.” Nature 553: 96–100. 10.1038/nature25167. 29258294

[acel70055-bib-0119] Miller, B. C. , D. R. Sen , R. al Abosy , et al. 2019. “Subsets of Exhausted CD8(+) T Cells Differentially Mediate Tumor Control and Respond to Checkpoint Blockade.” Nature Immunology 20: 326–336. 10.1038/s41590-019-0312-6. 30778252 PMC6673650

[acel70055-bib-0120] Mohanty, R. , C. Chowdhury , S. Arega , P. Sen , P. Ganguly , and N. Ganguly . 2019. “CAR T Cell Therapy: A New Era for Cancer Treatment (Review).” Oncology Reports 42: 2183–2195. 10.3892/or.2019.7335. 31578576

[acel70055-bib-0121] Montero, J. C. , S. Seoane , A. Ocaña , and A. Pandiella . 2011. “Inhibition of SRC Family Kinases and Receptor Tyrosine Kinases by Dasatinib: Possible Combinations in Solid Tumors.” Clinical Cancer Research 17: 5546–5552. 10.1158/1078-0432.Ccr-10-2616. 21670084

[acel70055-bib-0122] Morgan, R. A. , L. A. Johnson , J. L. Davis , et al. 2012. “Recognition of Glioma Stem Cells by Genetically Modified T Cells Targeting EGFRvIII and Development of Adoptive Cell Therapy for Glioma.” Human Gene Therapy 23: 1043–1053. 10.1089/hum.2012.041. 22780919 PMC3472555

[acel70055-bib-0123] Mustjoki, S. , K. Auvinen , A. Kreutzman , et al. 2013. “Rapid Mobilization of Cytotoxic Lymphocytes Induced by Dasatinib Therapy.” Leukemia 27: 914–924. 10.1038/leu.2012.348. 23192016

[acel70055-bib-0124] Nabrinsky, E. , J. Macklis , and J. Bitran . 2022. “A Review of the Abscopal Effect in the Era of Immunotherapy.” Cureus 14: e29620. 10.7759/cureus.29620. 36321062 PMC9604762

[acel70055-bib-0125] Nishikawa, H. , and S. Sakaguchi . 2014. “Regulatory T Cells in Cancer Immunotherapy.” Current Opinion in Immunology 27: 1–7. 10.1016/j.coi.2013.12.005. 24413387

[acel70055-bib-0126] Onorati, A. , A. P. Havas , B. Lin , et al. 2022. “Upregulation of PD‐L1 in Senescence and Aging.” Molecular and Cellular Biology 42: e0017122. 10.1128/mcb.00171-22. 36154662 PMC9583718

[acel70055-bib-0127] Ostrand‐Rosenberg, S. , and P. Sinha . 2009. “Myeloid‐Derived Suppressor Cells: Linking Inflammation and Cancer.” Journal of Immunology 182: 4499–4506. 10.4049/jimmunol.0802740. PMC2810498 19342621

[acel70055-bib-0128] Pant, A. , and M. Lim . 2023. “CAR‐T Therapy in GBM: Current Challenges and Avenues for Improvement.” Cancers (Basel) 15: 1249. 10.3390/cancers15041249. 36831591 PMC9954019

[acel70055-bib-0129] Pardoll, D. M. 2012. “The Blockade of Immune Checkpoints in Cancer Immunotherapy.” Nature Reviews. Cancer 12: 252–264. 10.1038/nrc3239. 22437870 PMC4856023

[acel70055-bib-0130] Pauken, K. E. , and E. J. Wherry . 2015. “Overcoming T Cell Exhaustion in Infection and Cancer.” Trends in Immunology 36: 265–276. 10.1016/j.it.2015.02.008. 25797516 PMC4393798

[acel70055-bib-0131] Phuphanich, S. , C. J. Wheeler , J. D. Rudnick , et al. 2013. “Phase I Trial of a Multi‐Epitope‐Pulsed Dendritic Cell Vaccine for Patients With Newly Diagnosed Glioblastoma.” Cancer Immunology, Immunotherapy 62: 125–135. 10.1007/s00262-012-1319-0. 22847020 PMC3541928

[acel70055-bib-0132] Ponnappan, S. , and U. Ponnappan . 2011. “Aging and Immune Function: Molecular Mechanisms to Interventions.” Antioxidants & Redox Signaling 14: 1551–1585. 10.1089/ars.2010.3228. 20812785 PMC3061194

[acel70055-bib-0133] Poorebrahim, M. , J. Melief , Y. Pico de Coaña , S. L. Wickström , A. Cid‐Arregui , and R. Kiessling . 2021. “Counteracting CAR T Cell Dysfunction.” Oncogene 40: 421–435. 10.1038/s41388-020-01501-x. 33168929 PMC7808935

[acel70055-bib-0134] Posey, A. D., Jr. , R. M. Young , and C. H. June . 2024. “Future Perspectives on Engineered T Cells for Cancer.” Trends in Cancer 10: 687–695. 10.1016/j.trecan.2024.05.007. 38853073

[acel70055-bib-0135] Prasanna, P. G. , D. E. Citrin , J. Hildesheim , et al. 2021. “Therapy‐Induced Senescence: Opportunities to Improve Anticancer Therapy.” Journal of the National Cancer Institute 113: 1285–1298. 10.1093/jnci/djab064. 33792717 PMC8486333

[acel70055-bib-0136] Quail, D. F. , and J. A. Joyce . 2013. “Microenvironmental Regulation of Tumor Progression and Metastasis.” Nature Medicine 19: 1423–1437. 10.1038/nm.3394. PMC3954707 24202395

[acel70055-bib-0137] Quail, D. F. , and J. A. Joyce . 2017. “The Microenvironmental Landscape of Brain Tumors.” Cancer Cell 31: 326–341. 10.1016/j.ccell.2017.02.009. 28292436 PMC5424263

[acel70055-bib-0138] Rafiq, S. , C. S. Hackett , and R. J. Brentjens . 2020. “Engineering Strategies to Overcome the Current Roadblocks in CAR T Cell Therapy.” Nature Reviews Clinical Oncology 17: 147–167. 10.1038/s41571-019-0297-y. PMC7223338 31848460

[acel70055-bib-0139] Rao, S. G. , and J. G. Jackson . 2016. “SASP: Tumor Suppressor or Promoter? Yes!” Trends Cancer 2: 676–687. 10.1016/j.trecan.2016.10.001. 28741506

[acel70055-bib-0140] Reimann, M. , J. Schrezenmeier , P. Richter‐Pechanska , et al. 2021. “Adaptive T‐Cell Immunity Controls Senescence‐Prone MyD88‐ Or CARD11‐Mutant B‐Cell Lymphomas.” Blood 137: 2785–2799. 10.1182/blood.2020005244. 33232972

[acel70055-bib-0141] Ribas, A. , R. Dummer , I. Puzanov , et al. 2017. “Oncolytic Virotherapy Promotes Intratumoral T Cell Infiltration and Improves Anti‐PD‐1 Immunotherapy.” Cell 170: 1109–1119. 10.1016/j.cell.2017.08.027. 28886381 PMC8034392

[acel70055-bib-0142] Roberson, R. S. , S. J. Kussick , E. Vallieres , S. Y. Chen , and D. Y. Wu . 2005. “Escape From Therapy‐Induced Accelerated Cellular Senescence in p53‐Null Lung Cancer Cells and in Human Lung Cancers.” Cancer Research 65: 2795–2803. 10.1158/0008-5472.Can-04-1270. 15805280

[acel70055-bib-0143] Rohaan, M. W. , S. Wilgenhof , and J. Haanen . 2019. “Adoptive Cellular Therapies: The Current Landscape.” Virchows Archiv 474: 449–461. 10.1007/s00428-018-2484-0. 30470934 PMC6447513

[acel70055-bib-0144] Rojas, L. A. , Z. Sethna , K. C. Soares , et al. 2023. “Personalized RNA Neoantigen Vaccines Stimulate T Cells in Pancreatic Cancer.” Nature 618: 144–150. 10.1038/s41586-023-06063-y. 37165196 PMC10171177

[acel70055-bib-0145] Rong, L. , N. Li , and Z. Zhang . 2022. “Emerging Therapies for Glioblastoma: Current State and Future Directions.” Journal of Experimental & Clinical Cancer Research 41: 142. 10.1186/s13046-022-02349-7. 35428347 PMC9013078

[acel70055-bib-0146] Rourke, D. M. , M. L. P. Nasrallah , A. Desai , et al. 2017. “A Single Dose of Peripherally Infused EGFRvIII‐Directed CAR T Cells Mediates Antigen Loss and Induces Adaptive Resistance in Patients With Recurrent Glioblastoma.” Science Translational Medicine 9: eaaa0984. 10.1126/scitranslmed.aaa0984. 28724573 PMC5762203

[acel70055-bib-0147] Rudolph, K. L. , S. Chang , H. W. Lee , et al. 1999. “Longevity, Stress Response, and Cancer in Aging Telomerase‐Deficient Mice.” Cell 96: 701–712. 10.1016/s0092-8674(00)80580-2. 10089885

[acel70055-bib-0148] Rufer, N. , M. Migliaccio , J. Antonchuk , R. K. Humphries , E. Roosnek , and P. M. Lansdorp . 2001. “Transfer of the Human Telomerase Reverse Transcriptase (TERT) Gene Into T Lymphocytes Results in Extension of Replicative Potential.” Blood 98, no. 3: 597–603. 10.1182/blood.v98.3.597. 11468156

[acel70055-bib-0149] Ruhland, M. K. , A. J. Loza , A. H. Capietto , et al. 2016. “Stromal Senescence Establishes an Immunosuppressive Microenvironment That Drives Tumorigenesis.” Nature Communications 7: 11762. 10.1038/ncomms11762. PMC4899869 27272654

[acel70055-bib-0150] Ruscetti, M. , J. Leibold , M. J. Bott , et al. 2018. “NK Cell‐Mediated Cytotoxicity Contributes to Tumor Control by a Cytostatic Drug Combination.” Science 362: 1416–1422. 10.1126/science.aas9090. 30573629 PMC6711172

[acel70055-bib-0151] Ruscetti, M. , J. P. Morris IV , R. Mezzadra , et al. 2020. “Senescence‐Induced Vascular Remodeling Creates Therapeutic Vulnerabilities in Pancreas Cancer.” Cell 181: 424–441. 10.1016/j.cell.2020.03.008. 32234521 PMC7278897

[acel70055-bib-0152] Russell, S. J. , K. W. Peng , and J. C. Bell . 2012. “Oncolytic Virotherapy.” Nature Biotechnology 30: 658–670. 10.1038/nbt.2287. PMC3888062 22781695

[acel70055-bib-0153] Salam, R. , A. Saliou , F. Bielle , et al. 2023. “Cellular Senescence in Malignant Cells Promotes Tumor Progression in Mouse and Patient Glioblastoma.” Nature Communications 14: 441. 10.1038/s41467-023-36124-9. PMC9883514 36707509

[acel70055-bib-0154] Saleh, T. , V. J. Carpenter , L. Tyutyunyk‐Massey , et al. 2020. “Clearance of Therapy‐Induced Senescent Tumor Cells by the Senolytic ABT‐263 via Interference With BCL‐X(L) ‐BAX Interaction.” Molecular Oncology 14: 2504–2519. 10.1002/1878-0261.12761. 32652830 PMC7530780

[acel70055-bib-0155] Saleki, K. , and N. Rezaei . 2022. Handbook of Cancer and Immunology, 1–29. Springer International Publishing.

[acel70055-bib-0156] Sasidharan Nair, V. , R. Saleh , S. M. Toor , F. S. Cyprian , and E. Elkord . 2021. “Metabolic Reprogramming of T Regulatory Cells in the Hypoxic Tumor Microenvironment.” Cancer Immunology, Immunotherapy 70: 2103–2121. 10.1007/s00262-020-02842-y. 33532902 PMC8289790

[acel70055-bib-0157] Saxena, M. , S. H. van der Burg , C. J. M. Melief , and N. Bhardwaj . 2021. “Therapeutic Cancer Vaccines.” Nature Reviews. Cancer 21: 360–378. 10.1038/s41568-021-00346-0. 33907315

[acel70055-bib-0158] Scharping, N. E. , A. V. Menk , R. S. Moreci , et al. 2016. “The Tumor Microenvironment Represses T Cell Mitochondrial Biogenesis to Drive Intratumoral T Cell Metabolic Insufficiency and Dysfunction.” Immunity 45: 374–388. 10.1016/j.immuni.2016.07.009. 27496732 PMC5207350

[acel70055-bib-0159] Scheffel, M. J. , G. Scurti , M. M. Wyatt , et al. 2018. “N‐Acetyl Cysteine Protects Anti‐Melanoma Cytotoxic T Cells From Exhaustion Induced by Rapid Expansion via the Downmodulation of Foxo1 in an Akt‐Dependent Manner.” Cancer Immunology, Immunotherapy 67: 691–702. 10.1007/s00262-018-2120-5. 29396710 PMC5862784

[acel70055-bib-0160] Schito, L. , and G. L. Semenza . 2016. “Hypoxia‐Inducible Factors: Master Regulators of Cancer Progression.” Trends Cancer 2: 758–770. 10.1016/j.trecan.2016.10.016. 28741521

[acel70055-bib-0161] Schmitt, C. A. , B. Wang , and M. Demaria . 2022. “Senescence and Cancer — Role and Therapeutic Opportunities.” Nature Reviews Clinical Oncology 19: 619–636. 10.1038/s41571-022-00668-4. PMC9428886 36045302

[acel70055-bib-0162] Shahbandi, A. , F. Y. Chiu , N. A. Ungerleider , et al. 2022. “Breast Cancer Cells Survive Chemotherapy by Activating Targetable Immune‐Modulatory Programs Characterized by PD‐L1 or CD80.” Nature Cancer 3: 1513–1533. 10.1038/s43018-022-00466-y. 36482233 PMC9923777

[acel70055-bib-0163] Sharma, P. , A. Aaroe , J. Liang , and V. K. Puduvalli . 2023. “Tumor Microenvironment in Glioblastoma: Current and Emerging Concepts.” Neurooncology Advances 5: vdad009. 10.1093/noajnl/vdad009. PMC10034917 36968288

[acel70055-bib-0164] Sharma, P. , and J. P. Allison . 2015a. “Immune Checkpoint Targeting in Cancer Therapy: Toward Combination Strategies With Curative Potential.” Cell 161: 205–214. 10.1016/j.cell.2015.03.030. 25860605 PMC5905674

[acel70055-bib-0165] Sharma, P. , and J. P. Allison . 2015b. “The Future of Immune Checkpoint Therapy.” Science 348: 56–61. 10.1126/science.aaa8172. 25838373

[acel70055-bib-0166] Slaets, H. , N. Veeningen , P. L. J. de Keizer , N. Hellings , and S. Hendrix . 2024. “Are Immunosenescent T Cells Really Senescent?” Aging Cell 23, no. 10: e14300. 10.1111/acel.14300. 39113243 PMC11464117

[acel70055-bib-0167] Solomon, I. , M. Amann , A. Goubier , et al. 2020. “CD25‐T(Reg)‐Depleting Antibodies Preserving IL‐2 Signaling on Effector T Cells Enhance Effector Activation and Antitumor Immunity.” Nature Cancer 1: 1153–1166. 10.1038/s43018-020-00133-0. 33644766 PMC7116816

[acel70055-bib-0168] Sullivan, D. , and E. L. Pearce . 2015. “Targeting T Cell Metabolism for Therapy.” Trends in Immunology 36: 71–80. 10.1016/j.it.2014.12.004. 25601541 PMC4323623

[acel70055-bib-0169] Sullivan, E. A. , R. Wallis , F. Mossa , and C. L. Bishop . 2024. “The Paradox of Senescent‐Marker Positive Cancer Cells: Challenges and Opportunities.” NPJ Aging 10: 41. 10.1038/s41514-024-00168-y. 39277623 PMC11401916

[acel70055-bib-0170] Takasugi, M. , Y. Yoshida , and N. Ohtani . 2022. “Cellular Senescence and the Tumour Microenvironment.” Molecular Oncology 16: 3333–3351. 10.1002/1878-0261.13268. 35674109 PMC9490140

[acel70055-bib-0171] Thomas, A. A. , M. S. Ernstoff , and C. E. Fadul . 2012. “Immunotherapy for the Treatment of Glioblastoma.” Cancer Journal 18: 59–68. 10.1097/PPO.0b013e3182431a73. 22290259 PMC3269657

[acel70055-bib-0172] Thomas, R. , G. al‐Khadairi , J. Roelands , et al. 2018. “NY‐ESO‐1 Based Immunotherapy of Cancer: Current Perspectives.” Frontiers in Immunology 9: 947. 10.3389/fimmu.2018.00947. 29770138 PMC5941317

[acel70055-bib-0173] Todo, T. , H. Ito , Y. Ino , et al. 2022. “Intratumoral Oncolytic Herpes Virus G47∆ for Residual or Recurrent Glioblastoma: A Phase 2 Trial.” Nature Medicine 28: 1630–1639. 10.1038/s41591-022-01897-x. PMC9388376 35864254

[acel70055-bib-0174] Togashi, Y. , K. Shitara , and H. Nishikawa . 2019. “Regulatory T Cells in Cancer Immunosuppression ‐ Implications for Anticancer Therapy.” Nature Reviews. Clinical Oncology 16: 356–371. 10.1038/s41571-019-0175-7. 30705439

[acel70055-bib-0175] Topalian, S. L. , C. G. Drake , and D. M. Pardoll . 2015. “Immune Checkpoint Blockade: A Common Denominator Approach to Cancer Therapy.” Cancer Cell 27: 450–461. 10.1016/j.ccell.2015.03.001. 25858804 PMC4400238

[acel70055-bib-0176] Toso, A. , A. Revandkar , D. di Mitri , et al. 2014. “Enhancing Chemotherapy Efficacy in Pten‐Deficient Prostate Tumors by Activating the Senescence‐Associated Antitumor Immunity.” Cell Reports 9: 75–89. 10.1016/j.celrep.2014.08.044. 25263564

[acel70055-bib-0177] Turner, S. J. , T. J. Bennett , and N. L. La Gruta . 2021. “CD8(+) T‐Cell Memory: The Why, the When, and the How.” Cold Spring Harbor Perspectives in Biology 13: 38661. 10.1101/cshperspect.a038661. PMC8091951 33648987

[acel70055-bib-0178] Vander Heiden, M. G. , L. C. Cantley , and C. B. Thompson . 2009. “Understanding the Warburg Effect: The Metabolic Requirements of Cell Proliferation.” Science 324: 1029–1033. 10.1126/science.1160809. 19460998 PMC2849637

[acel70055-bib-0179] Verma, N. K. , B. H. S. Wong , Z. S. Poh , et al. 2022. “Obstacles for T‐Lymphocytes in the Tumour Microenvironment: Therapeutic Challenges, Advances and Opportunities Beyond Immune Checkpoint.” eBioMedicine 83: 104216. 10.1016/j.ebiom.2022.104216. 35986950 PMC9403334

[acel70055-bib-0180] Waickman, A. T. , and J. D. Powell . 2012. “mTOR, Metabolism, and the Regulation of T‐Cell Differentiation and Function.” Immunological Reviews 249: 43–58. 10.1111/j.1600-065X.2012.01152.x. 22889214 PMC3419491

[acel70055-bib-0181] Wang, L. , L. Lankhorst , and R. Bernards . 2022b. “Exploiting Senescence for the Treatment of Cancer.” Nature Reviews Cancer 22: 340–355. 10.1038/s41568-022-00450-9. 35241831

[acel70055-bib-0182] Wang, T. W. , Y. Johmura , N. Suzuki , et al. 2022a. “Blocking PD‐L1‐PD‐1 Improves Senescence Surveillance and Ageing Phenotypes.” Nature 611: 358–364. 10.1038/s41586-022-05388-4. 36323784

[acel70055-bib-0183] Watowich, M. B. , M. R. Gilbert , and M. Larion . 2023. “T Cell Exhaustion in Malignant Gliomas.” Trends in Cancer 9: 270–292. 10.1016/j.trecan.2022.12.008. 36681605 PMC10038906

[acel70055-bib-0184] Weber, R. , V. Fleming , X. Hu , et al. 2018. “Myeloid‐Derived Suppressor Cells Hinder the Anti‐Cancer Activity of Immune Checkpoint Inhibitors.” Frontiers in Immunology 9: 1310. 10.3389/fimmu.2018.01310. 29942309 PMC6004385

[acel70055-bib-0185] Weller, M. , and E. Le Rhun . 2019. “Immunotherapy for Glioblastoma: Quo Vadis?” Nature Reviews Clinical Oncology 16: 405–406. 10.1038/s41571-019-0195-3. 30867572

[acel70055-bib-0186] Wen, P. Y. , D. A. Reardon , T. S. Armstrong , et al. 2019. “A Randomized Double‐Blind Placebo‐Controlled Phase II Trial of Dendritic Cell Vaccine ICT‐107 in Newly Diagnosed Patients With Glioblastoma.” Clinical Cancer Research 25: 5799–5807. 10.1158/1078-0432.Ccr-19-0261. 31320597 PMC8132111

[acel70055-bib-0187] Wherry, E. J. 2011. “T Cell Exhaustion.” Nature Immunology 12: 492–499. 10.1038/ni.2035. 21739672

[acel70055-bib-0188] Wherry, E. J. , and M. Kurachi . 2015. “Molecular and Cellular Insights Into T Cell Exhaustion.” Nature Reviews. Immunology 15: 486–499. 10.1038/nri3862. PMC4889009 26205583

[acel70055-bib-0189] Widodo, S. S. , M. Dinevska , L. M. Furst , S. S. Stylli , and T. Mantamadiotis . 2021. “IL‐10 in Glioma.” British Journal of Cancer 125: 1466–1476. 10.1038/s41416-021-01515-6. 34349251 PMC8609023

[acel70055-bib-0190] Wilkinson, E. 2023. “UK‐BioNTech Partnership for mRNA Cancer Vaccines.” Lancet Oncology 24: 846. 10.1016/s1470-2045(23)00339-x. 37454666

[acel70055-bib-0191] Woroniecka, K. I. , K. E. Rhodin , P. Chongsathidkiet , K. A. Keith , and P. E. Fecci . 2018. “T‐Cell Dysfunction in Glioblastoma: Applying a New Framework.” Clinical Cancer Research 24: 3792–3802. 10.1158/1078-0432.Ccr-18-0047. 29593027 PMC6095741

[acel70055-bib-0192] Xia, L. , L. Oyang , J. Lin , et al. 2021. “The Cancer Metabolic Reprogramming and Immune Response.” Molecular Cancer 20: 28. 10.1186/s12943-021-01316-8. 33546704 PMC7863491

[acel70055-bib-0193] Xu, M. , T. Pirtskhalava , J. N. Farr , et al. 2018. “Senolytics Improve Physical Function and Increase Lifespan in Old Age.” Nature Medicine 24: 1246–1256. 10.1038/s41591-018-0092-9. PMC6082705 29988130

[acel70055-bib-0194] Yamanaka, R. , T. Abe , N. Yajima , et al. 2003. “Vaccination of Recurrent Glioma Patients With Tumour Lysate‐Pulsed Dendritic Cells Elicits Immune Responses: Results of a Clinical Phase I/II Trial.” British Journal of Cancer 89: 1172–1179. 10.1038/sj.bjc.6601268. 14520441 PMC2394324

[acel70055-bib-0195] Yan, X. , N. Imano , K. Tamaki , M. Sano , and K. Shinmura . 2021. “The Effect of Caloric Restriction on the Increase in Senescence‐Associated T Cells and Metabolic Disorders in Aged Mice.” PLoS One 16: e0252547. 10.1371/journal.pone.0252547. 34143796 PMC8213184

[acel70055-bib-0196] Young, R. M. , N. W. Engel , U. Uslu , N. Wellhausen , and C. H. June . 2022. “Next‐Generation CAR T‐Cell Therapies.” Cancer Discovery 12: 1625–1633. 10.1158/2159-8290.Cd-21-1683. 35417527 PMC9262817

[acel70055-bib-0197] Yu, P.‐J. , M. Zhou , Y. Liu , and J. Du . 2025. “Senescent T Cells in Age‐Related Diseases.” Aging and Disease 16: 321–344. 10.14336/ad.2024.0219. PMC11745454 38502582

[acel70055-bib-0198] Zamora, A. E. , J. C. Crawford , and P. G. Thomas . 2018. “Hitting the Target: How T Cells Detect and Eliminate Tumors.” Journal of Immunology 200: 392–399. 10.4049/jimmunol.1701413. PMC6116355 29311380

[acel70055-bib-0199] Zhang, H. , S. Li , D. Wang , et al. 2024. “Metabolic Reprogramming and Immune Evasion: The Interplay in the Tumor Microenvironment.” Biomarker Research 12: 96. 10.1186/s40364-024-00646-1. 39227970 PMC11373140

[acel70055-bib-0200] Zhang, J. , T. He , L. Xue , and H. Guo . 2021. “Senescent T Cells: A Potential Biomarker and Target for Cancer Therapy.” eBioMedicine 68: 103409. 10.1016/j.ebiom.2021.103409. 34049248 PMC8170103

[acel70055-bib-0201] Zhao, J. , A. X. Chen , R. D. Gartrell , et al. 2019. “Immune and Genomic Correlates of Response to Anti‐PD‐1 Immunotherapy in Glioblastoma.” Nature Medicine 25: 462–469. 10.1038/s41591-019-0349-y. PMC6810613 30742119

[acel70055-bib-0202] Zhao, Y. , Q. Shao , and G. Peng . 2020. “Exhaustion and Senescence: Two Crucial Dysfunctional States of T Cells in the Tumor Microenvironment.” Cellular & Molecular Immunology 17: 27–35. 10.1038/s41423-019-0344-8. 31853000 PMC6952436

[acel70055-bib-0203] Zingoni, A. , F. Antonangeli , S. Sozzani , A. Santoni , M. Cippitelli , and A. Soriani . 2024. “The Senescence Journey in Cancer Immunoediting.” Molecular Cancer 23: 68. 10.1186/s12943-024-01973-5. 38561826 PMC10983694

